# Peer network approaches for improving HIV testing, prevention and care utilisation among men in sub-Saharan Africa: a scoping review

**DOI:** 10.1136/bmjopen-2025-106124

**Published:** 2026-03-12

**Authors:** Wilfred Ouma Otambo, Guy Harling, Maxime Inghels, Margot Otto, Ntombifuthi Blose, Khai Hoan Tram, Frank Tanser, Paul Mee

**Affiliations:** 1 South Africa Centre of Excellence in Epidemiological Modelling and Analysis, Centre for Epidemic Response and Innovation, School of Data Science and Computational Thinking, Stellenbosch University, Stellenbosch, South Africa; 2 Institute for Global Health, University College London, London, UK; 3 Africa Health Research Institute, KwaZulu-Natal, South Africa; 4 School of Public Health, Faculty of Health Sciences, University of the Witwatersrand, Johannesburg, South Africa; 5 School of Nursing & Public Health, University of KwaZulu-Natal, Durban, South Africa; 6 Lincoln Institute for Rural and Coastal Health, College of Health and Science, University of Lincoln, Lincoln, UK; 7 Division of Allergy & Infectious Diseases, Department of Medicine, University of Washington, Washington, DC, USA

**Keywords:** HIV & AIDS, EPIDEMIOLOGY, Public health, Epidemiology

## Abstract

**Abstract:**

**Introduction:**

Limited male engagement in HIV prevention and care is a global challenge more pronounced in sub-Saharan Africa (SSA) contributing to ongoing transmission. However, peer network interventions show promise in improving engagement.

**Objective:**

To map and synthesise evidence on peer network interventions for HIV prevention and care among men in SSA, with a focus on the types of strategies used, populations reached and how these interventions address cultural, social and structural barriers across the HIV care cascade.

**Design:**

Scoping review of peer-reviewed literature, conducted and reported in accordance with the Preferred Reporting Items for Systematic Reviews and Meta-Analyses extension for Scoping Reviews.

**Setting:**

Community and facility-based HIV prevention and care settings across multiple countries in SSA.

**Participants:**

Populations included men having sex with men, men in key occupational groups (fishermen, truck drivers), adolescents and young people, and men living with HIV. Studies not conducted in SSA, not peer-reviewed or not focused on male peer networks were excluded.

**Interventions:**

Peer network interventions included peer education, peer navigation, HIV self-testing (HIVST) distribution, adherence support groups, disclosure and stigma-reduction counselling, technology-enabled peer support (SMS and social media) and community-based antiretroviral therapy (ART) delivery. Intervention duration and intensity varied across studies.

**Outcome measures:**

Primary outcomes included HIV testing uptake, linkage to care, ART initiation, adherence, retention in care and viral suppression. Secondary outcomes included stigma reduction, disclosure and engagement among hard-to-reach male populations.

**Methods:**

We conducted a review of literature published between November 2013 and November 2024, searching PubMed, Web of Science, Scopus and Cochrane databases. Studies were included if they examined peer network approaches in HIV prevention and care among men in SSA.

**Results:**

A total of 905 records were identified, of which 75 studies met the inclusion criteria. Peer network interventions were implemented across diverse SSA contexts and male populations. Strategies such as peer-led education, social diffusion models, HIVST distribution and technology-enabled peer support consistently improved HIV testing uptake, linkage to care, ART initiation and adherence. Contextually tailored interventions such as community-based outreach addressing occupational risk environments and economic vulnerabilities were particularly effective in engaging men traditionally underserved by facility-based services. However, challenges persisted, including variable linkage to care following HIVST and sensitivity to user costs.

**Conclusions:**

Peer-led interventions in HIV care for men in SSA effectively address cultural, social and structural barriers, improving testing, ART adherence and viral suppression. Tailored, technology-enhanced and community-based approaches ensure equitable HIV prevention and treatment outcomes, despite challenges like linkage to care.

**STRENGTHS AND LIMITATIONS OF THIS STUDY**

- The scoping review followed established methodological guidance such as the Arksey and O’Malley framework and Preferred Reporting Items for Systematic Reviews and Meta-Analyses extension for Scoping Reviews reporting standards.
- A comprehensive search strategy was implemented across multiple databases.
- The review was restricted to English-language, peer-reviewed studies, which might have excluded relevant evidence from non-English-speaking settings.
- Grey literature and reports were not systematically searched, potentially underrepresenting implementation-focused interventions.

## Introduction

 In the ongoing global response to HIV, great progress has been made in improving treatment, prevention and awareness.^1^ However, limited engagement of men in HIV prevention and care is a global challenge, and it is pronounced in sub-Saharan Africa (SSA), where male engagement with HIV care has been persistently lower than women.^2^,^4^ Reasons for this disparity are driven by cultural, social and structural barriers, including entrenched gender norms that discourage seeking healthcare, confidentiality concerns, HIV-related stigma and the perception that services are predominantly women-centred.^2 5^ Additionally, men in SSA often face access barriers, such as work-related time constraints and limited access to male-friendly services.^6^,^9^ These challenges disproportionately affect men at high risk of HIV acquisition, including marginalised populations such as men having sex with men (MSM), further exacerbating disparities in HIV testing and care engagement.^10 11^ This highlights the importance of developing tailored interventions that address men’s specific social and structural constraints, reduce stigma, promote testing and improve sustained engagement in care, particularly in underserved rural and high-risk areas.

Peer and social networks interventions have emerged as promising tools in public health, leveraging social influence to shape health behaviours, reduce stigma and improve access to care.^5 12 13^ In SSA, peer networks have facilitated HIV prevention and management by engaging communities, promoting HIV self-testing (HIVST)^514^,^17^ and enhancing knowledge and behaviours, particularly among youth and people living with HIV (PLHIV).^18^,^23^ Despite their recognised potential, a systematic understanding of the scope, characteristics and outcome of peer network approaches specifically targeting men remains limited.^3 5^ Evidence on the extent, implementation strategy and reported outcomes across diverse populations and geographic contexts is limited, creating a critical knowledge gap that impedes efforts to optimise HIV care for men and achieve equitable prevention and treatment outcomes.^3 24^

To address this gap, the current study aims to systematically map the scope and characteristics of peer network interventions targeting men in SSA, describe the strategies employed, and identify reported opportunities and challenges across diverse contexts.^21 23^ As a scoping review, this approach is intended to comprehensively map existing evidence rather than assess intervention effectiveness, thereby accommodating heterogeneity in study designs, populations and outcomes, and informing future research and policy priorities.

This scoping review examines peer network interventions in HIV care for men in SSA, by mapping their geographic and population coverage, describing intervention strategies and delivery mode, and summarising reported outcomes related to HIV testing, linkage to care, treatment initiation and retention. The review further explores how these interventions address cultural, social and structural barriers to care, with the aim of informing equitable and contextually appropriate HIV programming for men across the region.

## Methods

### Framework for conducting the scoping review

This scoping review was conducted in accordance with the methodological framework proposed by Arksey and O’Malley, further refined by Levac *et al*,^25 26^ and further guided by the Joanna Briggs Institute recommendations for scoping reviews.^27^,^30^ Reporting followed the Preferred Reporting Items for Systematic Reviews and Meta-Analyses extension for Scoping Reviews (PRISMA-ScR) checklist to ensure transparent and comprehensive presentation of methods and findings.^31 32^ Together, these frameworks and guidelines informed the identification and clarification of the research question, the design and conduct of the systematic search, study selection using an iterative, team-based approach, structured data charting, and the descriptive and thematic synthesis of evidence

### Search strategy and information sources

To identify relevant studies, we used keywords and controlled vocabulary terms related to “Peer network”, “HIV testing”, “HIV treatment”, “HIV prevention”, “Men” and “Africa”. These keyword searches and subject headings were combined with Boolean operators (OR and AND) and truncation (*) to locate relevant peer-reviewed literature on peer network interventions to HIV care among men in SSA.

Database searches were conducted in PubMed, Scopus, Web of Science and Cochrane (CENTRAL) with full search equation in all the databases (online supplemental table 1). Database searches were supplemented with searching on Google Scholar as well as forward and backward citation searches of relevant articles. Grey literature (including conference proceedings, programme reports, policy documents, trial registries and organisational websites) was not systematically searched. This decision was made due to feasibility constraints and the objective of focusing on peer-reviewed evidence. The implications of this are acknowledged in the Limitations section. Searches were continually updated to identify and incorporate the most up-to-date evidence where appropriate up to November 2024.

### Eligibility criteria

Articles were included in this review if they met the following inclusion criteria: (1) were conducted in at least one SSA country; (2) evaluated male-focused peer or social network intervention addressing HIV testing, treatment or prevention; (3) Published in peer-reviewed journals; (4) were published between November 2013 and November 2024 and (5) Published in English. The start date of November 2013 was selected to capture contemporary peer network interventions implemented in the context of expanded antiretroviral therapy (ART) eligibility, HIVST scale-up and differentiated service delivery models that have increasingly shaped HIV programming over the past decade.

The exclusion criteria included: articles were those not meeting these inclusion criteria, such as non-peer-reviewed studies, those conducted outside SSA, or those not addressing male peer networks in HIV care or not addressing HIV-related outcomes, studies published in languages other than English. Language restrictions were applied due to feasibility constraints; consequently, relevant studies published in French or Portuguese may not have been captured, particularly from Francophone and Lusophone SSA countries.

### Protocol registration

The protocol has been registered with PROSPERO under the registration number CRD42024553230 and is publicly available at https://www.crd.york.ac.uk/PROSPERO/recorddashboard

### Screening and selection of sources

Initial screening of titles and abstracts was done using Rayyan application software (a web-based systematic review screening platform) to identify relevant articles in the database.^33^ Two reviewers independently screened all titles and abstracts against the eligibility criteria. Rayyan was used to manage records, remove duplicates, blind reviewers during initial screening and flag conflicts.

A full-text review of selected articles was conducted independently by the same two reviewers to confirm eligibility. Discrepancies at both stages were resolved through discussion and consensus, with involvement of a third reviewer where necessary. Reasons for exclusion of irrelevant articles were documented and reported in the PRISMA flow diagram.

### Data charting process

Data were charted using a structured extraction form developed specifically for this study, informed by prior scoping reviews of network and HIV interventions. The form was pilot tested to ensure consistency and clarity. The form captured study characteristics, intervention details and outcomes. Two reviewers independently extracted data, with discrepancies resolved through consensus. Data were organised into predefined themes aligned with the HIV care cascade (testing, linkage to care, ART initiation, adherence) and network intervention domains (identification, segmentation, induction, restructuring). No additional data were sought from investigators.

### Data items

Variables extracted included study setting, population characteristics, intervention design, network strategies, outcomes along HIV care cascade and relevant contextual factors. Assumptions were made that reported outcomes reflected intervention impacts as reported by the authors. Diverse interventions were categorised into the four network domains for consistency.

### Critical appraisal of individual sources

Consistent with the objective of a scoping review, no formal methodological quality or risk-of-bias assessment was conducted. This approach aligns with the PRISMA-ScR guidelines^31^ which emphasise mapping the breadth and nature of available evidence rather than evaluating study quality.

### Analytical framework for network interventions in HIV care cascade

The analysis was guided by an Analytical Framework for Network Intervention in the HIV Care Cascade, integrating network theory with HIV service delivery stages. Drawing on established network intervention models,^34 35^ studies were examined across four domains: (a) identification of key nodes, focusing on how influential individuals or groups were selected; (b) segmentation and contextual adaptation, examining the targeting of subgroups and tailoring of interventions; (c) induction and behaviour propagation, assessing mechanisms for diffusing health-promoting behaviour and (d) dynamic network restructuring and equity, exploring efforts to modify social network structures to improve access to care and promoting equity.

### Synthesis of results

Data extraction involved systematically organising information obtained from studies into predefined themes to capture the range of peer network interventions and their outcomes.^1^ Data from studies were systematically extracted and mapped to the HIV care cascade, assessing impacts on testing, linkage to care, ART initiation and adherence.

The extracted data were then mapped to the HIV care cascade, examining how interventions affected outcomes like testing, linkage to care, ART initiation and adherence.

Thematic and comparative analysis highlighted common and unique intervention strategies, with attention to contextual factors such as stigma, gender norms and economic barriers that influenced outcomes. The analytical framework involved focusing on the roles of peer networks in promoting HIV care outcomes, addressing stigma and improving ART adherence. Comparative analysis highlighted both common strategies and unique adaptations.

### Patient and public involvement

Patients and members of the public were not involved in the design, conduct, or reporting of this scoping review, as it was based on the analysis of previously published literature.

## Results

**Figure 1:**
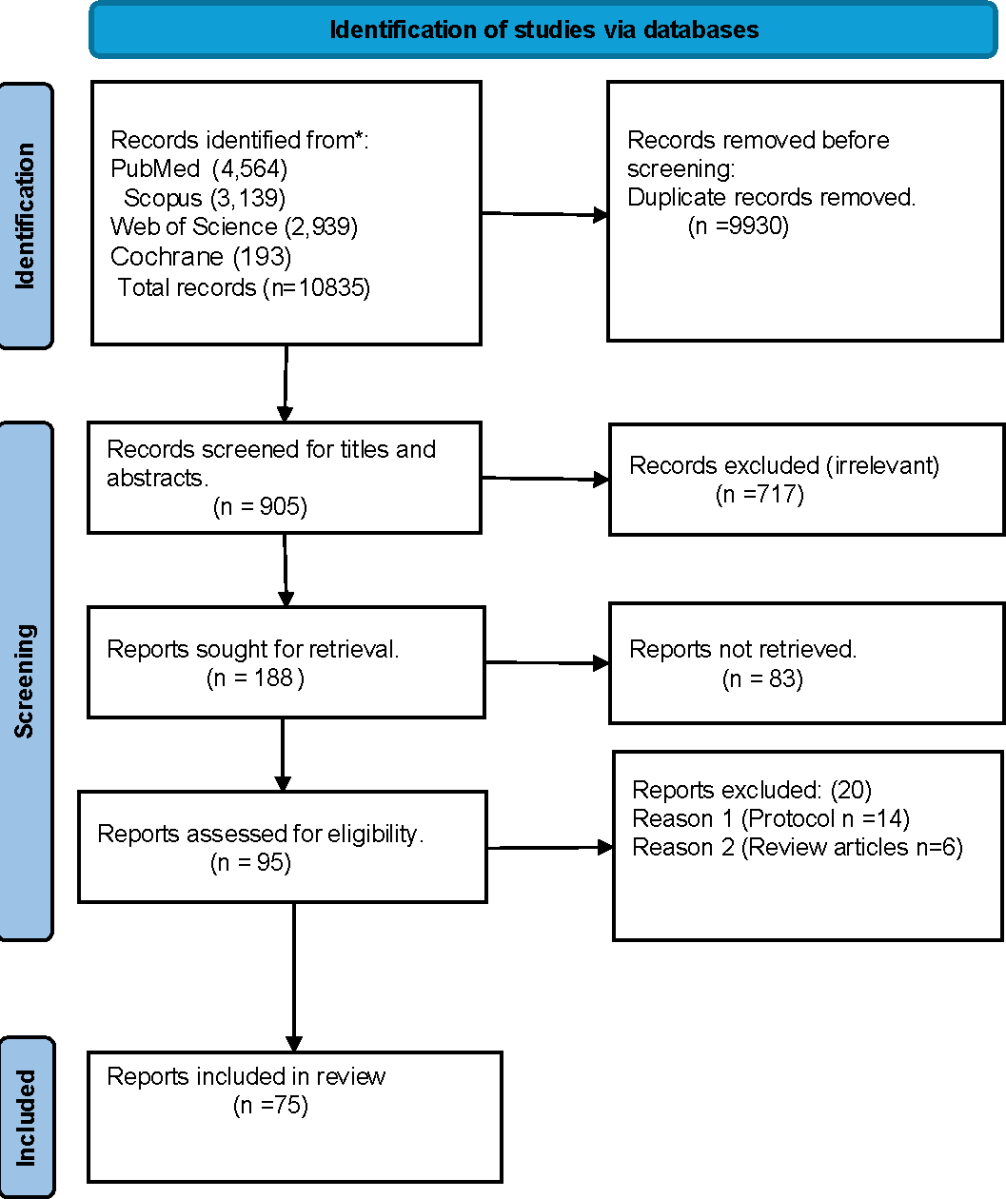
PRISMA flow chart of study selection. PRISMA, Preferred Reporting Items for Systematic Reviews and Meta-Analyses.

The database searches yielded 10 835 articles, with 905 unique records. After screening titles and abstracts for inclusion criteria, 188 articles were selected for full-text review. Of these, 95 met all inclusion criteria; however, 20 were excluded as review or protocol articles. This resulted in a final set of 75 articles included in the review. The selection process is detailed in a PRISMA flow diagram (figure 1). Summary of Peer Network Interventions in HIV Care Among Men in SSA is summarised in online supplemental table 2

### Characteristics of sources of evidence

The 75 included studies were SSA countries such as Uganda, Tanzania, South Africa, Nigeria and Zimbabwe, focusing on diverse populations including MSM,^1016 36^,^38^ fishermen,^2339^,^41^ truck drivers,^42 43^ youth and occupational groups.^44^ Data charted included study location, target population, intervention type (eg, peer-led education, HIVST distribution, adherence clubs), strategies (eg, technology use, community outreach), outcomes (eg, testing rates, ART adherence, viral suppression) and contextual factors (eg, stigma, gender norms).

### Network intervention strategies used

Network intervention strategies of all four types were identified (table 1). Peer network interventions effectively identified key nodes to optimise reach, leveraging social diffusion principles to promote behaviour change and overcome barriers. Peer educators and navigators played pivotal roles in promoting behaviour change and ART adherence within networks, facilitating viral load suppression and prevention strategies.^3 18 45^ Segmentation and contextual adaptation were evident in Zimbabwe, where peer education engaged youth and community-specific activities, such as soccer and vocational training, involved men in prevention efforts.^46 47^ Induction and behaviour propagation mechanisms, including group-based approaches, increased testing uptake, ART adherence and awareness of self-testing and pre-exposure prophylaxis (PrEP) in Tanzania, Uganda and South Africa.^44 48 49^ Dynamic network restructuring and equity were demonstrated through the use of gatekeeping roles, which improved engagement, enhanced access to care and strengthened intervention effectiveness.^50^

**Table 1:** Domains of network intervention strategies

Category	Description	Example applications and outcome	Reference
Identification of key nodes	Identifying influential individuals or groups within the network to maximise reach and impact.	Peer educators improved linkage to ART services (Uganda, Nigeria), promoted behaviour change and ART adherence and reached youth effectively (Zimbabwe). Treatment Ambassadors used peer counselling to overcome ART barriers (South Africa) Peer leaders engaged underserved populations, increasing testing and ART adherence (Uganda, Tanzania) and promoted gender-based violence discussions (Tanzania) Community leaders facilitated stigma reduction and education (Zimbabwe)	5 55 56 75 82 83
Segmentation and contextual adaptation	Dividing the network into subgroups based on shared characteristics and tailoring interventions to meet the specific needs of each subgroup.	Adherence clubs targeted people living with HIV to enhance ART adherence (South Africa). Peer education was highly effective in communicating health messages to youth (Zimbabwe). Soccer and vocational training, adapted to cultural and age-specific interests successfully engaged men in prevention efforts (South Africa). Fishermen received customised community-based approaches to address unique risks in the sex-for-fish economy (Kenya). Men having sex with men, (MSM) benefited from outreach campaigns tailored to stigmatised communities (Côte d'Ivoire, Mali and Senegal). Adolescent-focused peer education integrated holistic health messaging with entrepreneurial training, improving testing and mental well-being (Zimbabwe).	39 46 47 66 67
Induction and behaviour propagation	Encouraging desired behaviours (eg, safer sexual practices, ART adherence) to spread through peer influence within the network.	Group-based approaches helped in overcoming stigma, enhanced social cohesion and provided tailored support in underserved populations (South Africa). Community-based peer education improved awareness, testing and linkage to care (Tanzania, Zimbabwe). Peer leaders in fishing communities distributed HIVST kits and linked individuals to care (Uganda). Peer-driven models increased PrEP adherence among Gay, Bisexual and Other Cisgender MSM (Ghana). Compassion-focused therapy fostered stigma reduction and social diffusion of safer practices (Uganda). Peer networks expanded HIV prevention strategies, increasing testing and condom use among key populations (Ghana). Peer-based education and advocacy successfully promoted condom use (South Africa, Ghana). HIV testing rates increased through cascading peer influence.	10 23 46 48 57 84 85
Dynamic network restructuring and equity	Modifying existing network structures to enhance healthcare access, equity and communication within marginalised groups.	Gatekeeping roles significantly influence engagement and intervention effectiveness. Peer educators working with PWID in Kenya overcame social isolation and stigma, improving HIV and hepatitis C care (Kenya). Peer health leaders positioned as change agents within networks drove discussions on gender norms and HIV prevention (Tanzania). Peer outreach ensured equitable distribution of HIVST kits, reaching first-time testers in underserved (Côte d’Ivoire, Mali and Senegal). A mobile technology-based intervention informed by behavioural economics to improve ART adherence among youth (Uganda).	36 53 59 63 68

* Network intervention strategies, category, description, example application and outcome.

ART, antiretroviral therapy; HIVST, HIV self-testing; PrEP, pre-exposure prophylaxis; PWID, persons who inject drugs.

### Impact of peer network characteristics and strategies on HIV care

Table 2 summarises the diversity of peer network approaches and their impact on HIV care. Target peer networks varied widely, including PLHIV,^45 51 52^ MSM,^1016 36^,^38^ persons who inject drugs,^53^ fishing communities,^2339^,^41^ urban and rural populations,^38 53 54^ and occupational groups like mountain climbing porters^44^ and truck drivers.^42 43^ Peer network strategies included peer-led education, social influence models, peer-nominated leaders, peer support groups, peer-driven health campaigns, peer-to-peer networks, social media engagement and HIVST kit distribution through peers. The recruitment of participants relied on predefined peer groups, such as adherence clubs or community-based organisations, structured engagement within specific populations and dynamic recruitment methods such as respondent-driven sampling. Peer-led education sessions, including both individual and group-based formats, were effective in enhancing HIV knowledge, reducing stigma and normalising testing and treatment uptake. Peer navigation, where trained peers assisted individuals in navigating healthcare systems, ensured linkage to care and retention in treatment programmes. Distribution of HIVST kits, condoms and PrEP was disseminated by peers, reaching underserved populations. Peer network interventions in HIV care significantly improved ART adherence, HIV testing rates and stigma reduction across diverse populations. Strategies like peer-led education, HIVST distribution and tailored cultural approaches effectively addressed barriers, particularly among underserved groups such as MSM, adolescents and fishermen (table 2).

**Table 2:** Diversity of peer network approaches and impact on HIV care

Targeted populations	Methods of leveraging peer networks	Recruitment strategies	Types of interventions delivered	Observed outcomes	References
People living with HIV	Peer-led educational sessions, one-on-one mentoring, sharing ART experiences and treatment as prevention messaging and disclosure support.	Community-based engagement, voluntary participation, CHW-supported follow-up.	ART initiation and adherence support, stigma reduction through counselling.	Improved ART adherence and initiation, enhanced treatment literacy, viral suppression awareness and improved disclosure.	3 15 86 87
Men having sex with men	Social influence models; peer education; network-based HIV prevention and adherence strategies, including HIVST kit distribution, SMS and SMS+ peer navigation for linkage to care.	Respondent-driven sampling, peer nomination and social network outreach, mobile-based SMS recruitment.	Condom promotion, PrEP promotion, ART adherence, stigma reduction and HIVST promotion, linkage to care and ART initiation support.	Increased: condom use, PrEP adherence, higher testing rates and reduction in internalised stigma, improved ART initiation rates with SMS+ peer navigation.	16 17 54 58 69 71 77
Adolescents and young people	Peer education with holistic integration, network-based behaviour change and targeted ART adherence sessions, youth peer mentors employed as part of clinic staff.	School-based recruitment, community outreach, clinic-based mentorship programmes.	Sexual health education, peer-to-peer counselling, support for disclosure and ART adherence interventions.	Improved mental well-being, higher ART adherence and testing rates, and better psychosocial outcomes, and higher viral suppression.	46 65
Persons who inject with drugs	Peer navigators and educators provide navigation services to reduce access barriers to HIV and hepatitis C care.	Targeted outreach in urban and rural settings, recruitment at harm reduction centres.	Modified social ecological model addressing barriers in care, stigma reduction and harm reduction education.	Increased access to care, reduced stigma and enhanced treatment retention.	53
Male mountain porters	Peer networks targeting hard-to-reach populations through satisfaction-based HIVST promotion.	Community-based peer nomination.	HIV self-testing promotion, satisfaction-based follow-ups.	Increased HIVST uptake and satisfaction.	44
Truck drivers	Choice-based HIVST interventions combining oral and blood-based testing options.	Recruitment at truck stops, clinics near highways and community events.	Distribution of HIV self-test kits, peer-led health discussions and linkage to care support.	Increased testing rates and preferences for oral-based testing.	42 43
Social network ‘camp’ members	Peer influence using microfinance integration.	Social network-based peer leadership.	Microfinance training combined with gender-equitable discussions.	Improved ART adherence and gender norm perceptions.	50 63
Urban and rural men	Mobile-based and home-based HIV testing campaigns.	Community-wide recruitment and incentivised door-to-door testing.	ART initiation, linkage to care and HIV prevention messaging.	Increased ART initiation and reduction in HIV incidence.	88 89
Fishing community members	Community-based peer approaches focusing on contextual risks like transactional sex, ‘sex-for-fish’, peer-nominated leaders providing oral HIVST kits.	Selection of trusted community members, fishing villages and community gatherings.	HIVST promotion and linkage to care, peer-led discussions about extramarital partnerships, spousal communication and behaviour change related to financial pressures.	High HIVST acceptability and linkage to care, enhanced spousal communication, improved ART initiation and risk reduction.	23 39 41 48 90
Men in soccer and vocational training	Soccer-based and vocational peer groups addressing HIV prevention.	Soccer club and vocational training participation.	Male-centred, interest-based HIV prevention.	Higher adherence to ART and engagement in HIV prevention.	47
Men at alcohol-HIV risk	Multilevel peer interventions addressing individual and community-level risks associated with alcohol consumption and HIV.	Recruitment at drinking establishments through community facilitators.	Individual workshops, community activation and risk reduction education targeting masculinity-related barriers.	Increased condom use, more HIV prevention discussions and stronger perceptions of safer sex norms.	64 77
Urban and high-traffic areas	Peer networks offering mobile HIV testing and U=U (Undetectable=Untransmittable) messaging.	Recruitment in urban hotspots such as markets, bus stations and mobile clinics.	Peer educators delivering U=U messages and facilitating HIV testing.	Increased testing uptake and reduced stigma related to HIV testing.	54 57
Men in low-income or high-unemployment areas	Peer-based treatment ambassador programmes and ART adherence interventions.	Recruitment from community spaces and clinics targeting vulnerable populations.	Home-based ART delivery, patient navigation skills training and supportive counselling focusing on economic considerations.	Increased ART engagement, higher viral suppression rates and reduced caregiver burden.	55 83

Diversity of peer network approaches and their impact on HIV care.

ART, antiretroviral therapy; CHW, Community Health Worker; HIVST, HIV self-testing; PrEP, pre-exposure prophylaxis.

### Peer network approaches and HIV care outcomes

Table 3 summarises the peer network strategies linked to outcomes and the countries where these strategies were implemented. Peer network approaches employed diverse strategies to engage peers in HIV prevention, treatment and adherence, addressing cultural, social and structural barriers. These strategies include peer education sessions for prevention in Uganda,^17^ behaviour change among fishermen in Kenya,^39^ enhanced HIV knowledge and self-testing uptake among hard-to-reach populations in Tanzania,^44^ and improved medication adherence and viral suppression in Nigeria and South Africa.^45 55^

**Table 3:** Peer network approaches and HIV care outcomes

Category	Peer network strategies	Description	Linked outcomes	Countries implemented
Peer education	Peer-led educational sessions, mentoring	Socioecological framework-based sessions focusing on ART initiation and adherence	Higher ART initiation and adherence; increased viral suppression; peer social support identified as a key facilitator of engagement	Malawi, Uganda, South Africa, Eswatini^3^
	Peer-based HIV prevention advocacy	Group sessions (Game Changers) and mentorship	Increased condom use, reduced stigma, higher social network density and safer sexual practices	Uganda^17^, South Africa^17 77 85^
	Behavioural change interventions using peer facilitators	Change agents disseminating HIV knowledge	Increased HIV knowledge, improved service engagement and safer behaviour; and reduced dropouts in social network-based interventions	Tanzania^56^, Kenya, ^53^, South Africa^54^
	Youth-led or peer-led initiatives	Youth-led peer education delivering health messages and holistic interventions	Increased service uptake, improved mental well-being and higher HIV knowledge	Zimbabwe^46^, Tanzania^90^
	Peer-driven ART initiation and adherence support	One-on-one mentoring for PWHIV	Improved ART initiation and adherence, with viral suppression achieved among disengaged populations	South Africa^55 75^
	Peer-based structural and community-level interventions	Peer educators addressing stigma, barriers to care and access to prevention resources	Addressed stigma, improved service navigation and adherence, and enhanced care through structural and community-level interventions	Kenya^53^, Ghana^10^,
HIV testing and treatment	Peer-led HIV self-testing (HIVST) distribution	Peer educators distributing HIVST kits	High HIVST uptake, increased first-time testers and effective linkage to care	Côte d'Ivoire, Mali, Senegal^67^, South Africa^51^,
	Peer-to-peer navigation	Peer navigators and social networks promoting HIV testing, PrEP and ART adherence	Increased HIV testing and PrEP uptake, with successful linkage among never-testers	Côte d’Ivoire, Mali, Senegal^69^, Uganda^74^, South Africa^20^
	Community-based HIV testing interventions	Peer leaders in fishing communities	Increased testing uptake, high HIVST satisfaction and reduced gender gaps in testing	Tanzania^44 90^
	Differentiated service delivery models for ART	Adherence Clubs, pharmacy pick-up	Improved ART adherence, higher viral suppression and reduced stigma	South Africa^66^
	Youth-friendly participatory health projects	Peer educators supporting adolescent sexual and reproductive health initiatives	Sustained HIV testing and circumcision uptake despite reduced funding	Zimbabwe^82^
	HIV prevention strategies for MSM and other key populations	Peer educators facilitating HIV prevention (eg, condom use, PrEP adherence, stigma reduction)	Increased condom usage, reduced stigma and effective linkage to care for first-time testers	Ghana^10^, South Africa^51^
	HIV testing innovations for men	Community-wide campaigns, incentives or self-testing strategies to target male populations	Improved testing uptake, 28% reduced mortality (28%) with streamlined care	Kenya^42 52^, South Africa^49^
	Structural HIV prevention programmes	Soccer, vocational training and male-specific community education initiatives	Improved adherence, engagement and social cohesion; reduced HIV-related stigma; supported high-risk and disadvantaged groups	South Africa^47^, Botswana^91^
	Social network-based interventions	Leveraging peer and social network core members to promote testing and reduce stigma	Higher likelihood of testing in social network cores, improved norms around testing and reduced stigma	Tanzania^57 80^
Integration and utilisation of technology	Text-based interventions for adherence support	Messaging interventions targeting youth with adherence reminders, with or without peer-related adherence updates	Improved adherence with peer-linked SMS reminders; minimal effect with individual reminders alone	Uganda^59^
	Social media and mobile health (mHealth) approaches	SMART Connections and iCARE Nigeria promoting adherence	Increased HIV knowledge, acceptability of mHealth and potential scalability; limited impact on retention or social support	Nigeria^36 60^
	SMS-based interventions with peer navigation	SMS reminders with or without peer navigation	1.6-fold higher linkage to care and reduced time to ART initiation, and potential scalability	South Africa^71^
	Videoconference interventions for HIV risk and disclosure	Focused on status disclosure and risk reduction	High usability and satisfaction, with strong participant recommendations for use in wider implementation	South Africa^37^
	Peer support models	Dennis Peer Support to reduce stigma and improve linkage to care	Improved prevention and care engagement through emotional and informational peer support, and social networking	Ghana^16^
Stigma mitigation	Sociocentric peer influence on stigma	Peer network interactions shaping individual HIV stigma levels	Higher stigma clustered within stigmatising ties; peers living with HIV associated with lower stigma	Uganda^92^
	Peer support groups for stigma mitigation	Peer groups addressing internalised stigma among PLHIV	Reduced internalised stigma and narrower treatment gaps	Nigeria^15^
	Shikamana intervention for ART adherence	Nurse-led counselling and peer support for HIV-positive MSM	Increased ART adherence and viral suppression, with higher acceptability	Kenya^86^
	Participatory peer approaches to gender and HIV norms	Exploring the impact of social norms on sexual decision-making among youth	Complex and conflicting norms influenced youth HIV vulnerability	Zambia^78^
	Peer social network functions and structures	Support systems for MSM within social networks in urban and rural settings	Networks provided refuge, psychological support and fulfilled sexual needs	Ghana^19^
	Interactions of stigma, norms and care engagement	Influence of gender norms and stigma on HIV care adherence among fishermen	High stigma and inequitable norms linked to missed appointments and ARV doses; combined effects exacerbated treatment gaps	Uganda^72^
	NAMWEZA intervention for stigma reduction and care	Change agents	Significant reductions in stigma, depression; improved self-efficacy for safer sex	Tanzania^93^
	Youth-led peer mentor interventions	Training and compensating HIV-positive youth to mentor adolescents in clinics	Reduced stigma, improved ART adherence and viral suppression among young PLHIV	Zambia^65^
	Gender-transformative	Community mobilisation	Improved gender-equitable attitudes with limited immediate behavioural changes	South Africa^79^
Structural and community level	Community-driven HIV prevention and engagement strategies	Community-based initiatives: health campaigns, natural resource programmes and antiretroviral refill groups	Effective in increasing testing, reducing stigma and enhancing ART adherence	Senegal^94^, Zimbabwe^5^, Namibia^89^, Uganda^61^
	Peer-led outreach and HIV services for key populations	Enhanced peer outreach approaches targeting high-risk groups	Increased identification of new HIV-positive cases, improved ART adherence and effective engagement in HIV care	Burundi, Côte d’Ivoire, DRC^62^, Benue State, Nigeria^13^
	Combined microfinance and peer health leadership approaches	Peer-nominated leaders addressing gender norms, HIV prevention and IPV	Improved HIV testing rates and reduced inequitable gender norms	Tanzania^50 63^
	Peer network-based interventions for MSM	Social network strategies to strengthen healthcare services and outreach	Addressed MSM healthcare needs, improved collaboration among stakeholders and built a supportive platform for HIV care delivery	Kenya^11^
	Transition-focused peer support for re-entrants	Group-based behavioural interventions for men transitioning from incarceration	Improved ART adherence and engagement in care	South Africa^84^

Peer network strategies linked to outcomes and the countries where these strategies were implemented.

ART, antiretroviral therapy; IPV, Intimate Patner Violence; MSM, men having sex with men; PLHIV, people living with HIV; PrEP, pre-exposure prophylaxis.

Group-based interventions promoted behaviour change through social diffusion, whereby behaviour change initiated by a few individuals spread to others through network interactions across multiple settings including Uganda,^17 39 56^ Kenya and Tanzania. Peer networks have been key in the distribution of HIVST kits by overcoming stigma, improving clinic access, lowering costs, reaching first-time testers and encouraging behaviour change,^44 57 58^ with evidence from South Africa,^20^ Uganda^23^ and Kenya.^52^

Technology-enabled peer network interventions such as SMS, social media and smartphone-based support have enhanced ART adherence, retention in care and HIV-related knowledge^16 36 59 60^ as demonstrated in Ghana, Nigeria and Uganda.

Peer network-based community engagement, coupled with economic and social empowerment, has significantly improved HIV prevention and care, increased HIV testing, reduced gender norm inequities and improved treatment outcomes.^61 62^ In Tanzania, peer entrepreneurship has similarly contributed to reduction in gender norm inequities^63^ (table 3).

### Success and effectiveness of peer network interventions

Peer network interventions have improved HIV care by addressing barriers, promoting testing and enhancing treatment adherence (online supplemental table 2). Programmes across Africa, such as peer-led education in Tanzania and Ghana, tailored messaging in South Africa and initiatives like Uganda’s ‘Game Changer,’ have increased testing uptake and linkage to care.^17 44 56 64^ Peer-driven efforts in Zambia, Malawi and Eswatini supported ART adherence, among youth and men.^3 65^ Peer distribution of HIVST kits and differentiated care models like home ART delivery have reduced barriers and expanded access.^66^,^68^ Across diverse settings, peer mentorship and support have consistently improved testing, ART initiation and sustained adherence.^18 45 69^ Soccer and vocational training initiatives have improved male engagement in South Africa.^20 47^ Peer-based distribution of HIVST kits has helped identify ART-naive individuals previously missed by healthcare systems in Uganda and Kenya.^52 70^ Monetary incentives have increased testing uptake by 50% in South Africa, while lottery-based incentives addressed stigma and testing costs in Uganda.^49 70^ Differentiated service delivery models, such as home delivery of ART, have demonstrated success in reducing clinic-related barriers and achieving viral suppression, especially in South Africa.^20 71^ Peer-led educational programmes have influenced behaviour change in Ghana and Tanzania, increasing condom use and improving HIV knowledge.^10 56^ Peer educators have mitigated stigma by fostering open communication, providing psychosocial support and promoting health-seeking behaviours, especially among hard-to-reach groups.^72 73^ Despite challenges such as low linkage to care and demand sensitivity to costs, peer interventions remain vital in bridging gaps in HIV care, engaging marginalised populations and promoting sustainable outcomes in HIV prevention and treatment.^38 51^

### Impact of peer-led interventions on HIV care cascade

Peer-led interventions have effectively increased HIV testing across diverse settings through distribution of HIVST.^20 23 40 67 73 74^ Peer-driven initiatives have facilitated linkage to care through programmes such as ‘Game Changer’ in Uganda,^17^ and ‘Project YES’ in Zambia.^65^ Community-based approaches have facilitated ART initiation in marginalised groups.^3 52 75 76^ Interventions such as home delivery of ART and peer mentoring have significantly improved medication adherence and viral suppression.^45 47 59 60 65^ Table 4 summarises peer-led interventions in enhancing HIV testing, linkage to care, treatment initiation and adherence across diverse populations (table 4).

**Table 4:** Peer network and HIV care cascade

Cascade step	Description	Peer network interventions
HIV testing	Identifying individuals living with HIV through community outreach, self-testing kits or peer-led awareness campaigns.	Peer-led HIVST distribution in Côte d'Ivoire, Mali and Senegal, South Africa.^20 67 73^ Social network engagement for self-testing in Kenya, Undetectable=Untransmittable peer-delivered messaging increased testing in South Africa.^54^ Community-based HIVST intervention through peer-leader distribution in Uganda.^40^ Peer-driven HIV testing strategies to address the low testing strategy among MSM in Uganda.^74^ Peer-based distribution model of HIV self-test kits. Men distributors (seeds) recruited among male patients of a health facility, and among community members, offer HIVST kits to their peers in fishing communities in Uganda.^41^
Linkage to care	Ensuring individuals diagnosed with HIV are promptly connected to healthcare services for initial evaluations and ART enrolment.	HIVST distributed by peer leaders to social network members and linking first-time HIV-positive individuals to HIV care in Uganda.^23^ Peer leaders are involved in the testing and linkage tracking in Zambia.^95^ SMS and SMS with peer navigation for linkage to care in South Africa.^71^ Peer support for kit distribution and linkage to care in Uganda.^74^
Treatment initiation	Supporting newly diagnosed individuals to begin ART and addressing barriers to starting treatment.	Peer-led educational sessions with one-on-one mentoring and sharing successful experiences related to ART initiation and adherence in Malawi, Uganda, South Africa and Eswatini.^3^ Lay peer leadership boosts HIVST uptake and ART initiation within community settings among fishermen communities in Uganda.^40^ HIV testing and ART initiation for men with low CD4 in Kenya and Uganda.^52^ Peer-led educational sessions to improve ART initiation and adherence.^3^
Adherence	Promoting sustained engagement in ART through counselling, peer groups and addressing social or structural barriers to adherence.	Text-based intervention focused on adherence messages in Uganda.^59^ Social media platform for adherence, retention and support in Nigeria.^60^ Peer support linked to higher retention and treatment adherence, with increased viral suppression through increased social and emotional support in Kenya.^86^ Peer network for fisherfolk addressing gender norms and HIV stigma’s impact on treatment adherence in Uganda.^72^ Youth peer mentors serve as an effective, feasible approach for adolescent engagement in Zambia.^65^ Disclosure support improved trust, adherence and retention by providing safety and minimising negative outcomes in Nigeria.^13^

Peer network strategies linked to outcomes and the countries where these strategies were implemented.

ART, antiretroviral therapy; HIVST, HIV self-testing; MSM, men having sex with men.

## Discussion

Peer network approaches represent a promising strategy for improving HIV prevention, treatment and care among men in SSA. By using social connections, these interventions have successfully overcome cultural, social and structural barriers, reaching marginalised populations and encouraging behaviour change. Peer education and community mobilisation strategies such as peer-led discussions, group education sessions and targeted outreach have been associated with measurable improvements in HIV testing rates. For example, studies report increases in testing uptake ranging from 15% to 50% among men in fishing communities and key populations such as MSM and adolescents.^46 67^ Peer-led interventions, such as HIVST kit distribution, door-to-door campaigns and targeted messaging like ‘Undetectable=Untransmittable’, have facilitated first-time testing and improved knowledge of HIV status.^38 40 54^ These initiatives reduce stigma by promoting familiarity with testing, providing social proof and leveraging incentives.^70^ Integrating peer leadership with mobile health initiatives and community-based interventions has been linked to increased linkage to care and initiation of ART.^63 76^

Peer networks also contribute to improvements in treatment adherence and retention. Peer support groups, one-on-one mentoring and community-based education sessions have increased ART initiation and retention, with reported adherence improvements of 10%–30% in some studies.^3 17 65^ Peer educators and counsellors have been particularly effective in engaging men, youth and key populations who may otherwise be reluctant to access healthcare due to stigma, discrimination or fear of disclosure.^10 53 77^ Studies in Tanzania and Ghana show that peer-led behavioural change interventions have increased HIV knowledge, safer-sex behaviours and timely linkage to care, particularly among hard-to-reach populations.^44 56^ Differentiated service delivery models led by peers, such as home-based ART delivery and peer-assisted medication pick-ups, have also improved treatment retention and viral suppression.^66^ In Nigeria, peer-led initiatives have strengthened the continuum of care by addressing misinformation, stigma and structural barriers, demonstrating their potential in long-term treatment adherence.^45^

Technology-based peer interventions have further strengthened adherence and linkage to care by making HIV services more accessible and stigma-free. In South Africa and Nigeria, SMS-based peer navigation increased linkage to care 1.6-fold and reduced time to ART initiation.^71^ Smartphone-based peer networks provided ongoing motivation, emotional support and treatment reminders, highlighting the adaptability of peer-led interventions across technological landscapes.^59 60 71^ These platforms have been particularly effective in reaching remote, rural and socially isolated populations, demonstrating the adaptability of peer-led models to different technological landscapes.

Peer interventions that integrate economic empowerment strategies have had a profound impact on long-term HIV treatment adherence. Programmes combining peer health leadership with microfinance initiatives and vocational training have helped individuals overcome financial barriers to care and prioritise their health, showing positive effects on treatment adherence and long-term care engagement.^50 63^ Similarly, gender-transformative peer-led interventions have successfully engaged men in HIV care, challenged harmful gender norms and reduced barriers to testing and treatment, particularly among high-risk groups such as fishermen and young men.^72^

Peer-led interventions contribute to community-wide behaviour change by addressing gender-based violence, social stigma and negative attitudes towards HIV. In Zambia, peer support networks have helped youth engage in care and achieve viral suppression, while in Ghana and South Africa, community-led gender-transformative approaches have successfully tackled societal norms that hinder HIV prevention.^19 78 79^ Similarly, peer-led outreach programmes, including soccer-based and vocational training interventions, have improved treatment retention among adolescents and young adults.^47 55^

### Limitations

This scoping review has several limitations inherent to its design and conduct, consistent with guidance from the PRISMA-ScR.^31^ First, the review was limited to English-language, peer-reviewed publications, which may have resulted in the exclusion of studies published in other languages, particularly from Francophone SSA countries. Second, although a comprehensive search strategy was employed across multiple databases and supplemented by citation tracking and hand searching, grey literature, including programme reports, policy documents and unpublished evaluations, was not systematically searched. As a result, some community-based or implementation-focused peer network interventions may be under-represented. Third, in line with the objectives of a scoping review, no formal methodological quality appraisal or risk-of-bias assessment was conducted, as recommended by PRISMA-ScR for reviews aiming to map evidence rather than assess effectiveness. The findings should not be interpreted as estimates of intervention effectiveness or certainty of evidence. Fourth, the heterogeneity of study designs, intervention modalities, outcome measures and reporting quality limited direct comparability across studies and precluded quantitative synthesis. Outcomes were synthesised descriptively based on authors’ reports, which may vary in rigour and completeness.

### Implementation challenges of peer network intervention

Despite their effectiveness, peer network interventions face challenges that hinder their full potential. In South Africa, low linkage to care following HIVST and the reduced uptake of HIVST due to minimal fees in rural areas remain significant barriers.^38 51^ Stigma and discrimination deter men in Tanzania and Kenya from accessing services, even within trusted peer networks.^57 58^ Peer educators often lack adequate training and resources, affecting their ability to provide accurate information, especially around sensitive issues such as disclosure and stigma.^67 68^ Additionally, social dynamics and demographic factors, such as age and socioeconomic status, influence testing behaviour and uptake.^80 81^ Certain transient groups, such as mine workers and those in informal settlements, remain difficult to reach through peer networks.^20 55^ Sustainability is another challenge, as many peer-led initiatives rely on volunteerism and short-term funding, limiting their long-term impact.^49^ Moreover, technological barriers, such as digital literacy gaps and limited internet access, restrict the effectiveness of digital peer-led interventions in rural settings.^16 59^

### Research gaps

Several research gaps remain in understanding the full potential of peer-led interventions. While structured peer programmes, such as adherence clubs and sports-based interventions, have shown success,^39 46 47 66 67^ little is known about the role of informal peer interactions, such as casual conversations and social gatherings, in HIV-related decision-making and stigma reduction. In addition, peer-led interventions have been largely tailored to urban and rural settings,^38 53 54^ with limited research on their effectiveness in highly mobile populations, such as migrant workers, who are at increased risk of HIV infection.

The integration of digital tools into peer-led initiatives presents another research gap, particularly in rural areas with limited internet access. While digital peer engagement holds promise, further studies are needed to assess its effectiveness across different contexts. While short-term studies demonstrate improvements in testing and ART uptake,^51 76 79^ long-term sustainability of peer-led interventions remains unclear, with limited longitudinal data on whether peer networks maintain engagement over time or if their influence diminishes once structured support ends. Furthermore, little is known about the spillover effects of peer-led programmes whether men who participate in such interventions go on to influence others in their social circles, creating a broader culture of HIV awareness and care-seeking behaviour.

Cost-effectiveness studies are necessary to determine the financial viability of scaling up peer-led programmes in resource-limited settings. Improved tracking of linkage to care is also critical to assessing whether peer-based outreach translates into long-term retention in HIV services. Addressing these research gaps will be essential for refining peer-led approaches and ensuring their long-term impact on HIV prevention and treatment.

Expanding peer-driven interventions by integrating digital tools, scaling up economic empowerment programmes and strengthening peer mentorship initiatives will be critical in bridging gaps in HIV care and improving long-term health outcomes across SSA. To further address these challenges, the IMPERATIVE trial (ClinicalTrials.gov NCT06370923), a large-scale intervention leveraging male peer networks to enhance HIVST and PrEP uptake, is currently underway to improve men’s engagement in HIV care.

### Policy recommendation

To fully harness the potential of peer-led interventions, national HIV strategies should integrate and scale up peer network models, particularly in hard-to-reach and rural populations. Many peer-led initiatives remain donor-funded pilot projects with limited scalability. Increased government funding and structural support are necessary to institutionalise these models and sustain their long-term impact. Policymakers should also leverage technology, such as digital peer support platforms, SMS-based adherence reminders and mobile apps, to extend the reach of peer-led programmes, particularly in remote areas with limited healthcare access. Addressing harmful gender norms through peer-led gender-transformative interventions is crucial for engaging men, MSM and other marginalised groups in HIV care. Scaling up peer mentorship initiatives, integrating economic empowerment programmes and strengthening digital peer interventions will be essential for bridging gaps in HIV care and achieving long-term improvements in health outcomes across SSA.

## Conclusions

Peer network interventions have proven to be a successful tool in improving HIV prevention, treatment and care across SSA. By leveraging social connections and community-driven strategies, these interventions effectively reduce Stigma, increase HIV testing uptake and improve linkage to care and treatment adherence, particularly among hard-to-reach populations. Tailored peer-led strategies including HIVST distribution, mobile health initiatives, gender-transformative interventions and economic empowerment programmes have demonstrated ability to address structural and social barriers to care. Peer networks not only promote behaviour change but also foster trust, raise awareness and create supportive environments for HIV care. Digital platforms have further expanded the reach of these interventions, improving ART adherence and retention in care. However, challenges such as stigma, privacy concerns, limited training for peer educators and sustainability issues remain critical barriers to maximising their impact. Social and economic dynamics, including gender norms and financial instability, also influence intervention uptake and long-term engagement in care. To ensure the long-term success of peer-led interventions, sustained investment in research, funding and health system integration is essential. Strengthening these initiatives through technological innovations, policy-driven structural support and community-led adaptations will be key to addressing persistent gaps in the HIV care continuum.

## Supplementary material

10.1136/bmjopen-2025-106124 online supplemental table 1

## Data Availability

Data sharing not applicable as no datasets generated and/or analysed for this study. All data relevant to the study are included in the article or uploaded as supplementary information.

## References

[1] (2024). The urgency of now: aids at a crossroads. https://www.unaids.org/en/resources/documents/2024/global-aids-update-2024.

[2] Mashora MC (2020). Engaging men in HIV services in sub-Saharan Africa: an authors’ viewpoint on what has been done and what still needs to be done. Pan Afr Med J.

[3] Wiginton JM, Mathur S, Gottert A (2022). Hearing From Men Living With HIV: Experiences With HIV Testing, Treatment, and Viral Load Suppression in Four High-Prevalence Countries in Sub-Saharan Africa. Front Public Health.

[4] Hlongwa M, Mashamba-Thompson T, Makhunga S (2020). Men’s perspectives on HIV self-testing in sub-Saharan Africa: a systematic review and meta-synthesis. BMC Public Health.

[5] Mantell JE, Masvawure TB, Mapingure M (2019). Engaging men in HIV programmes: a qualitative study of male engagement in community-based antiretroviral refill groups in Zimbabwe. J Int AIDS Soc.

[6] George-Svahn L, Eriksson LE, Wiklander M (2021). Barriers to HIV testing as reported by individuals newly diagnosed with HIV infection in Sweden.

[7] Hlongwa M, Mashamba-Thompson T, Makhunga S (2020). Barriers to HIV testing uptake among men in sub-Saharan Africa: a scoping review. Afr J AIDS Res.

[8] Thapa S, Hannes K, Buve A (2018). Theorizing the complexity of HIV disclosure in vulnerable populations: A grounded theory study.

[9] Kumah E, Boakye DS, Boateng R (2023). Advancing the Global Fight Against HIV/Aids: Strategies, Barriers, and the Road to Eradication. Ann Glob Health.

[10] Abu-Ba’are GR, Shamrock OW, Apreku A (2023). Awareness and Willingness to use Condoms and Preexposure Prophylaxis among Gay, Bisexual, and Other Cisgendered Men who Have sex with men in Slum Communities in Ghana. BSGH-004. J Int Assoc Provid AIDS Care.

[11] van der Elst EM, Mudza R, Onguso JM (2020). A more responsive, multi‐pronged strategy is needed to strengthen HIV healthcare for men who have sex with men in a decentralized health system: qualitative insights of a case study in the Kenyan coast. J Intern AIDS Soc.

[12] Bhattacharjee P, Rego D, Musyoki H (2019). Evaluation of community-based HIV self-testing delivery strategies on reducing undiagnosed HIV infection, and improving linkage to prevention and treatment services, among men who have sex with men in Kenya: a programme science study protocol. BMC Public Health.

[13] Ibiloye O, Decroo T, van Olmen J (2023). Initial programme theory for community-based ART delivery for key populations in Benue State, Nigeria: a realist evaluation study. BMC Public Health.

[14] Harling G, Tsai AC (2019). Using Social Networks to Understand and Overcome Implementation Barriers in the Global HIV Response. J Acquir Immune Defic Syndr.

[15] Verinumbe T, Katomski A-S, Turpin G (2024). Characterizing the Relationship between HIV Peer Support Groups and Internalized Stigma Among People Living with HIV in Nigeria. AIDS Behav.

[16] Abubakari GM, Owusu-Dampare F, Ogunbajo A (2021). HIV Education, Empathy, and Empowerment (HIVE^3^): A Peer Support Intervention for Reducing Intersectional Stigma as a Barrier to HIV Testing among Men Who Have Sex with Men in Ghana. Int J Environ Res Public Health.

[17] Bogart LM, Matovu JKB, Wagner GJ (2020). A Pilot Test of Game Changers, a Social Network Intervention to Empower People with HIV to be Prevention Advocates in Uganda. AIDS Behav.

[18] Brashers DE, Basinger ED, Rintamaki LS (2017). Taking Control: The Efficacy and Durability of a Peer-Led Uncertainty Management Intervention for People Recently Diagnosed With HIV. Health Commun.

[19] Maina G, Strudwick G, Lalani Y (2018). Characterizing the Structure and Functions of Social Networks of Men Who Have Sex with Men in Ghana, West Africa: Implications for Peer-Based HIV Prevention. Journal of the Association of Nurses in AIDS Care.

[20] Adeagbo OA, Seeley J, Gumede D (2022). Process evaluation of peer-to-peer delivery of HIV self-testing and sexual health information to support HIV prevention among youth in rural KwaZulu-Natal, South Africa: qualitative analysis. BMJ Open.

[21] Conserve DF, Abu-Ba’are GR, Janson S Peer-based promotion and nurse-led distribution of hiv self-testing among networks of men in dar es salaam, tanzania: development and feasibility results of the step intervention. In Review.

[22] Ayala G, Sprague L, van der Merwe LL-A (2021). Peer- and community-led responses to HIV: A scoping review. PLoS ONE.

[23] Matovu JKB, Bogart LM, Nakabugo J (2020). Feasibility and acceptability of a pilot, peer-led HIV self-testing intervention in a hyperendemic fishing community in rural Uganda. PLoS ONE.

[24] Sharma M, Barnabas RV, Celum C (2017). Community-based strategies to strengthen men’s engagement in the HIV care cascade in sub-Saharan Africa. PLoS Med.

[25] Levac D, Colquhoun H, O’Brien KK (2010). Scoping studies: advancing the methodology. Implement Sci.

[26] Daudt HML, van Mossel C, Scott SJ (2013). Enhancing the scoping study methodology: a large, inter-professional team’s experience with Arksey and O’Malley’s framework. BMC Med Res Methodol.

[27] Khalil H, Peters M, Godfrey CM (2016). An Evidence‐Based Approach to Scoping Reviews. *Worldviews Ev Based Nurs*.

[28] Peters MDJ, Marnie C, Tricco AC (2020). Updated methodological guidance for the conduct of scoping reviews. *JBI Evid Synth*.

[29] Pollock D, Peters MDJ, Khalil H (2022). Recommendations for the extraction, analysis, and presentation of results in scoping reviews. JBI Evidence Synthesis.

[30] The Joanna Briggs Institute (2015). The Joanna Briggs Institute Reviewers’ Manual 2015: Methodology for JBI scoping reviews. Joanne Briggs Inst.

[31] Tricco AC, Lillie E, Zarin W (2018). PRISMA Extension for Scoping Reviews (PRISMA-ScR): Checklist and Explanation. Ann Intern Med.

[32] Peters MDJ, Marnie C, Colquhoun H (2021). Scoping reviews: reinforcing and advancing the methodology and application. Syst Rev.

[33] Ouzzani M, Hammady H, Fedorowicz Z (2016). Rayyan-a web and mobile app for systematic reviews. *Syst Rev*.

[34] Valente TW (2012). Network Interventions. Science.

[35] Robins G, Lusher D, Broccatelli C (2023). Multilevel network interventions: Goals, actions, and outcomes. Soc Networks.

[36] Kuhns LM, Johnson AK, Adetunji A (2021). Adaptation of evidence-based approaches to promote HIV testing and treatment engagement among high-risk Nigerian youth. PLoS ONE.

[37] Daniels J, Peters RPH, Portle S (2023). Developing the Speaking Out and Allying Relationships Intervention on Videoconference for HIV-Positive GBMSM in Eastern Cape, South Africa. Am J Mens Health.

[38] Chang W, Matambanadzo P, Takaruza A (2019). Effect of Prices, Distribution Strategies, and Marketing on Demand for HIV Self-testing in Zimbabwe: A Randomized Clinical Trial. *JAMA Netw Open*.

[39] Kwena ZA, Shisanya CA, Bukusi EA (2017). Jaboya (“Sex for Fish”): A Qualitative Analysis of Contextual Risk Factors for Extramarital Partnerships in the Fishing Communities in Western Kenya. Arch Sex Behav.

[40] Matovu JKB, Nambuusi A, Wanyenze RK (2021). Peer-leaders’ experiences and challenges in distributing HIV self-test kits in a rural fishing community, Rakai, Uganda. BMC Public Health.

[41] Choko AT, Nanfuka M, Birungi J (2018). A pilot trial of the peer-based distribution of HIV self-test kits among fishermen in Bulisa, Uganda. PLoS ONE.

[42] Kelvin EA, George G, Romo ML (2021). The Impact on HIV Testing Over 6 Months When Free Oral HIV Self-Test Kits Were Available to Truck Drivers in Kenya: A Randomized Controlled Trial. Front Public Health.

[43] Mantell JE, Khalifa A, Christian SN (2022). Preferences, beliefs, and attitudes about oral fluid and blood-based HIV self-testing among truck drivers in Kenya choosing not to test for HIV. Front Public Health.

[44] Njau B, Damian DJ, Lisasi E (2023). A theory-based behaviour change intervention to increase HIV Self-Testing uptake and linkage to HIV prevention, care and treatment for hard-to-reach populations in northern Tanzania. Tanzan J Health Res.

[45] Chime OH, Arinze-Onyia SU, Obionu CN (2018). Do peer support groups have an effect on medication adherence? A study among people living with HIV/AIDS in Enugu State, Nigeria. *Proceedings of Singapore Healthcare*.

[46] Mangombe A, Owiti P, Madzima B (2020). Does peer education go beyond giving reproductive health information? Cohort study in Bulawayo and Mount Darwin, Zimbabwe. BMJ Open.

[47] Rabie S, Bantjes J, Gordon S (2020). Who can we reach and who can we keep? Predictors of intervention engagement and adherence in a cluster randomized controlled trial in South Africa. BMC Public Health.

[48] Matovu JKB, Nambuusi A, Nakabirye S (2020). Formative research to inform the development of a peer-led HIV self-testing intervention to improve HIV testing uptake and linkage to HIV care among adolescents, young people and adult men in Kasensero fishing community, Rakai, Uganda: a qualitative study. BMC Public Health.

[49] Tanser FC, Kim H-Y, Mathenjwa T (2021). Home-Based Intervention to Test and Start (HITS): a community-randomized controlled trial to increase HIV testing uptake among men in rural South Africa. J Int AIDS Soc.

[50] Mulawa MI, Yamanis TJ, Kajula LJ (2018). Structural Network Position and Performance of Health Leaders Within an HIV Prevention Trial. AIDS Behav.

[51] Sithole N, Koole O, Sausi K (2022). Secondary Distribution of HIV Self-Testing Kits to Social and Sexual Networks of PLWH in KwaZulu-Natal, South Africa. A Brief Report. Front Public Health.

[52] Kamya MR, Petersen ML, Kabami J (2021). SEARCH Human Immunodeficiency Virus (HIV) Streamlined Treatment Intervention Reduces Mortality at a Population Level in Men With Low CD4 Counts. Clin Infect Dis.

[53] Ludwig-Barron NT, Guthrie BL, Mbogo L (2021). Barriers and facilitators of HIV and hepatitis C care among people who inject drugs in Nairobi, Kenya: a qualitative study with peer educators. Harm Reduct J.

[54] Smith P, Buttenheim A, Schmucker L (2021). Undetectable = Untransmittable (U = U) Messaging Increases Uptake of HIV Testing Among Men: Results from a Pilot Cluster Randomized Trial. AIDS Behav.

[55] Barnabas RV, Szpiro AA, Ntinga X (2022). Fee for home delivery and monitoring of antiretroviral therapy for HIV infection compared with standard clinic-based services in South Africa: a randomised controlled trial. Lancet HIV.

[56] Rewley J, Fawzi MCS, McAdam K (2020). Evaluating spillover of HIV knowledge from study participants to their network members in a stepped-wedge behavioural intervention in Tanzania. BMJ Open.

[57] Conserve DF, Issango J, Kilale AM (2019). Developing national strategies for reaching men with HIV testing services in Tanzania: results from the male catch-up plan. BMC Health Serv Res.

[58] Ndungu K, Gichangi P, Temmerman M (2023). Evaluation of factors associated with HIV self-testing Acceptability and Uptake among the MSM community in Nairobi, Kenya: A cross sectional study. *PLoS ONE*.

[59] MacCarthy S, Wagner Z, Mendoza-Graf A (2020). A randomized controlled trial study of the acceptability, feasibility, and preliminary impact of SITA (SMS as an Incentive To Adhere): a mobile technology-based intervention informed by behavioral economics to improve ART adherence among youth in Uganda. BMC Infect Dis.

[60] Dulli L, Ridgeway K, Packer C (2020). A Social Media-Based Support Group for Youth Living With HIV in Nigeria (SMART Connections): Randomized Controlled Trial. J Med Internet Res.

[61] Kabami J, Chamie G, Kwarisiima D (2017). Evaluating the feasibility and uptake of a community‐led HIV testing and multi‐disease health campaign in rural Uganda. J Int AIDS Soc.

[62] Lillie TA, Persaud NE, DiCarlo MC (2019). Reaching the unreached: Performance of an enhanced peer outreach approach to identify new HIV cases among female sex workers and men who have sex with men in HIV programs in West and Central Africa. PLoS ONE.

[63] Maman S, Mulawa MI, Balvanz P (2020). Results from a cluster-randomized trial to evaluate a microfinance and peer health leadership intervention to prevent HIV and intimate partner violence among social networks of Tanzanian men. PLoS ONE.

[64] Nelson LE, Wilton L, Agyarko-Poku T (2015). Predictors of Condom Use among Peer Social Networks of Men Who Have Sex with Men in Ghana, West Africa. PLoS ONE.

[65] Denison JA, Burke VM, Miti S (2020). Project YES! Youth Engaging for Success: A randomized controlled trial assessing the impact of a clinic-based peer mentoring program on viral suppression, adherence and internalized stigma among HIV-positive youth (15-24 years) in Ndola, Zambia. PLoS ONE.

[66] Mukumbang FC (2020). Leaving No Man Behind: How Differentiated Service Delivery Models Increase Men’s Engagement in HIV Care. *Int J Health Policy Manag*.

[67] Ky-Zerbo O, Desclaux A, Boye S (2022). “I take it and give it to my partners who will give it to their partners”: Secondary distribution of HIV self-tests by key populations in Côte d’Ivoire, Mali, and Senegal. BMC Infect Dis.

[68] Kra AK, Fotso AS, N’guessan KN (2022). Can HIV self-testing reach first-time testers? A telephone survey among self-test end users in Côte d’Ivoire, Mali, and Senegal. BMC Infect Dis.

[69] Eubanks A, Coulibaly B, Dembélé Keita B (2022). Socio-behavioral correlates of pre-exposure prophylaxis use and correct adherence in men who have sex with men in West Africa. BMC Public Health.

[70] Ndyabakira A, Getahun M, Byamukama A (2019). Leveraging incentives to increase HIV testing uptake among men: qualitative insights from rural Uganda. BMC Public Health.

[71] Lippman SA, de Kadt J, Ratlhagana MJ (2023). Impact of short message service and peer navigation on linkage to care and antiretroviral therapy initiation in South Africa. AIDS.

[72] Sileo KM, Wanyenze RK, Mukasa B (2021). The Intersection of Inequitable Gender Norm Endorsement and HIV Stigma: Implications for HIV Care Engagement for Men in Ugandan Fishing Communities. AIDS Behav.

[73] Kra AK, Colin G, Diop PM (2021). Introducing and Implementing HIV Self-Testing in Côte d’Ivoire, Mali, and Senegal: What Can We Learn From ATLAS Project Activity Reports in the Context of the COVID-19 Crisis?. Front Public Health.

[74] Okoboi S, Lazarus O, Castelnuovo B (2020). Peer distribution of HIV self-test kits to men who have sex with men to identify undiagnosed HIV infection in Uganda: A pilot study. PLoS ONE.

[75] Katz IT, Bogart LM, Fitzmaurice GM (2021). The Treatment Ambassador Program: A Highly Acceptable and Feasible Community-Based Peer Intervention for South Africans Living with HIV Who Delay or Discontinue Antiretroviral Therapy. AIDS Behav.

[76] Hayes R, Floyd S, Schaap A (2017). A universal testing and treatment intervention to improve HIV control: One-year results from intervention communities in Zambia in the HPTN 071 (PopART) cluster-randomised trial. PLoS Med.

[77] Kalichman SC, Simbayi LC, Cain D (2014). Randomized community-level HIV prevention intervention trial for men who drink in South African alcohol-serving venues. Eur J Public Health.

[78] Heslop J, Banda R (2013). Moving beyond the “male perpetrator, female victim” discourse in addressing sex and relationships for HIV prevention: peer research in Eastern Zambia. Reprod Health Matters.

[79] Pettifor A, Lippman SA, Gottert A (2018). Community mobilization to modify harmful gender norms and reduce hiv risk: results from a community cluster randomized trial in South Africa. J Intern AIDS Soc.

[80] Yamanis TJ, Dervisevic E, Mulawa M (2017). Social Network Influence on HIV Testing Among Urban Men in Tanzania. AIDS Behav.

[81] Hensen B, Lewis JJ, Schaap A (2015). Factors associated with HIV-testing and acceptance of an offer of home-based testing by men in rural Zambia. AIDS Behav.

[82] Mabaya S, Ncube R, Tweya H (2022). Retention and performance of peer educators and sustainability of HIV prevention services for adolescents in the Zimbabwe Smart-LyncAges project: an ecological study. Pan Afr Med J.

[83] Bogart LM, Phaladze N, Kgotlaetsile K (2024). Pilot Test of Mopati, a Multi-Level Adherence Intervention for People Living with HIV and Their Treatment Partners in Botswana. Int J Behav Med.

[84] An Y, Ntombela N, Hoffmann CJ (2022). “That makes me feel human”: a qualitative evaluation of the acceptability of an HIV differentiated care intervention for formerly incarcerated people re-entering community settings in South Africa. BMC Health Serv Res.

[85] Jemmott JB, Jemmott LS, O’Leary A (2014). Cluster-randomized controlled trial of an HIV/sexually transmitted infection risk-reduction intervention for South African men. Am J Public Health.

[86] Graham SM, Micheni M, Chirro O (2020). A Randomized Controlled Trial of the Shikamana Intervention to Promote Antiretroviral Therapy Adherence Among Gay, Bisexual, and Other Men Who Have Sex with Men in Kenya: Feasibility, Acceptability, Safety and Initial Effect Size. AIDS Behav.

[87] Lukyamuzi Z, Ssuna B, Mirembe RN (2023). Experiences and challenges of using community health worker-led mechanism in supporting HIV disclosure among adults living with HIV in heterosexual relationships in the rural Uganda. AIDS Res Ther.

[88] Lebelonyane R, Bachanas P, Block L (2021). To achieve 95-95-95 targets we must reach men and youth: High level of knowledge of HIV status, ART coverage, and viral suppression in the Botswana Combination Prevention Project through universal test and treat approach. PLoS ONE.

[89] Naidoo R, Johnson K (2013). Community-based conservation reduces sexual risk factors for HIV among men. Global Health.

[90] Matovu JKB, Mbita G, Hamilton A (2021). Men’s comfort in distributing or receiving HIV self-test kits from close male social network members in Dar Es Salaam, Tanzania: baseline results from the STEP project. BMC Public Health.

[91] Makhema J, Wirth KE, Pretorius Holme M (2019). Universal Testing, Expanded Treatment, and Incidence of HIV Infection in Botswana. N Engl J Med.

[92] Takada S, Nyakato V, Nishi A (2019). The social network context of HIV stigma: Population-based, sociocentric network study in rural Uganda. Social Science & Medicine.

[93] Smith Fawzi MC, Siril H, Liu Y (2019). Agents of change among people living with HIV and their social networks: stepped-wedge randomised controlled trial of the *NAMWEZA* intervention in Dar es Salaam, Tanzania. BMJ Glob Health.

[94] Dramé FM, Crawford EE, Diouf D (2013). A pilot cohort study to assess the feasibility of HIV prevention science research among men who have sex with men in Dakar, Senegal. J Int AIDS Soc.

[95] Kamanga J, Stankevitz K, Martinez A (2021). Improved HIV case finding among key populations after differentiated data driven community testing approaches in Zambia. PLoS ONE.

