# Supplementary material for: Peer network approaches for improving HIV testing, prevention and care utilisation among men in sub-Saharan Africa: a scoping review
Source: BMJ Open. 2026 Mar 12;16(3):e106124. doi: 10.1136/bmjopen-2025-106124 (PMC12983769; doi:10.1136/bmjopen-2025-106124)
Supplement: online supplemental table 1 [file bmjopen-16-3-s001.docx]

**Supplementary Table 1 Search Strategy for each database**

| **Data base** | **Syntax** |
| --- | --- |
| **PubMed**  (n=4,564) | ((((((((((((((((((((((((((((((((((((((((((((((((((((((((((((((peer network) OR (peer)) OR (network)) ) AND (HIV)) OR (HIV testing)) OR (HIV treatment)) OR (HIV prevention)) OR (HIV care)) OR (HIV management)) ) OR (HIV control))  AND  (Men)) OR (Man))  AND  (Africa)) OR (Angola)) OR (Benin)) OR (Botswana)) OR (Burkina Faso)) OR (Burundi)) OR (Cameroon)) OR (Cape Verde)) OR (Central African Rep)) OR (Chad)) OR (Comoro)) OR (Congo)) OR (Côte d'Ivoire)) OR (Djibouti)) OR (Eritrea)) OR (Eswatini)) OR (Swaziland)) OR (Ethiopia)) OR (Gabon)) OR (Gambia)) OR (Ghana)) OR (Guinea)) OR (Kenya)) OR (Lesotho)) OR (Liberia)) OR (Madagascar)) OR (Malawi)) OR (Mali)) OR (Mauritania)) OR (Mauritius)) OR (Mayotte)) OR (Mozambique)) OR (Namibia)) OR (Niger)) OR (Nigeria)) OR (Rwanda)) OR (São Tomé and Príncipe)) OR (Senegal)) OR (Seychelles)) OR (Sierra Leone)) OR (Somalia)) OR (South Africa)) OR (South Sudan)) OR (Sudan)) OR (Tanzania)) OR (Togo)) OR (Uganda)) OR (Zambia)) OR (Zimbabwe)  https://pubmed.ncbi.nlm.nih.gov/?term=%28%28%28%28%28%28%28%28%28%28%28%28%28%28%28%28%28%28%28%28%28%28%28%28%28%28%28%28%28%28%28%28%28%28%28%28%28%28%28%28%28%28%28%28%28%28%28%28%28%28%28%28%28%28%28%28%28%28%28%28%28%28peer+network%29+OR+%28peer%29%29+OR+%28network%29%29+%29+AND+%28HIV%29%29+OR+%28HIV+testing%29%29+OR+%28HIV+treatment%29%29+OR+%28HIV+prevention%29%29+OR+%28HIV+care%29%29+OR+%28HIV+management%29%29+%29+OR+%28HIV+control%29%29+AND+%28Men%29%29+OR+%28Man%29%29+AND+%28Africa%29%29+OR+%28Angola%29%29+OR+%28Benin%29%29+OR+%28Botswana%29%29+OR+%28Burkina+Faso%29%29+OR+%28Burundi%29%29+OR+%28Cameroon%29%29+OR+%28Cape+Verde%29%29+OR+%28Central+African+Rep%29%29+OR+%28Chad%29%29+OR+%28Comoro%29%29+OR+%28Congo%29%29+OR+%28Co%CC%82te+d%27Ivoire%29%29+OR+%28Djibouti%29%29+OR+%28Eritrea%29%29+OR+%28Eswatini%29%29+OR+%28Swaziland%29%29+OR+%28Ethiopia%29%29+OR+%28Gabon%29%29+OR+%28Gambia%29%29+OR+%28Ghana%29%29+OR+%28Guinea%29%29+OR+%28Kenya%29%29+OR+%28Lesotho%29%29+OR+%28Liberia%29%29+OR+%28Madagascar%29%29+OR+%28Malawi%29%29+OR+%28Mali%29%29+OR+%28Mauritania%29%29+OR+%28Mauritius%29%29+OR+%28Mayotte%29%29+OR+%28Mozambique%29%29+OR+%28Namibia%29%29+OR+%28Niger%29%29+OR+%28Nigeria%29%29+OR+%28Rwanda%29%29+OR+%28Sa%CC%83o+Tome%CC%81+and+Pri%CC%81ncipe%29%29+OR+%28Senegal%29%29+OR+%28Seychelles%29%29+OR+%28Sierra+Leone%29%29+OR+%28Somalia%29%29+OR+%28South+Africa%29%29+OR+%28South+Sudan%29%29+OR+%28Sudan%29%29+OR+%28Tanzania%29%29+OR+%28Togo%29%29+OR+%28Uganda%29%29+OR+%28Zambia%29%29+OR+%28Zimbabwe%29&filter=pubt.randomizedcontrolledtrial&filter=hum_ani.humans&filter=lang.english&filter=sex.male&filter=years.2013-2024 |
| **Web of Science**  (n= 2,939) | https://www.webofscience.com/wos/woscc/summary/f66aa16e-3027-4815-bfa2-f9bf908d3953-010db83095/relevance/1  ((((((((ALL=(peer network)) AND ALL=(HIV testing)) AND ALL=(HIV treatment )) AND ALL=(HIV prevention )) OR ALL=(HIV care )) OR ALL=(HIV management )) AND ALL=(men)) AND ALL=(Africa)) |
| **Scopus**  (n=3139) | peer AND network AND hiv AND treatment OR hiv AND testing OR hiv AND prevention AND men AND Africa |
| **Cochrane**  (n=193) | Peer network in Title Abstract Keyword AND HIV testing in Title Abstract Keyword OR HIV treatment in Title Abstract Keyword OR HIV prevention in Title Abstract Keyword AND men in Title Abstract Keyword - (Word variations have been searched) |

**Supplementary Table 2. Summary of Peer Network Interventions in HIV Care Among Men in Sub-Saharan Africa**

| **No** | **Study and Country** | **Study Design** | **Peer Group** | **Intervention Approach** | \| **Training & Support** \| \| --- \|  \|  \| \| --- \| | **Conceptual Understanding** | **Outcome** | **Challenges** | **Knowledge Gaps** | **Notes** |
| --- | --- | --- | --- | --- | --- | --- | --- | --- | --- | --- | --- | --- |
| 1 | (Wiginton et al., 2022)  Malawi, Uganda, South Africa, Eswatini | Cross-sectional | People Living HIV (PLHIV) | Peer-led educational sessions, one-on-one mentoring, sharing successful experiences related to ART initiation and adherence. | Supportive-directive counseling, education on treatment as prevention, viral suppression, and treatment literacy | Emphasizes social support, male-friendly services, counseling on ART benefits | Improved ART initiation, adherence, awareness, and viral suppression | Did not directly address stigma or masculinity-related barriers | Need for a better understanding of male engagement in HIV services across different contexts | Highlights socio-ecological approaches for male engagement in HIV care |
| 2 | (Bogart et al., 2020)  Uganda, | Randomized controlled Trial (RCT) | PLHIV | Game Changers, social influence model. Individuals with HIV act as HIV prevention advocates within their social network | Six session group session on compassion-focused therapy and cognitive behavior therapy. HIV-positive facilitators addressing internalized stigma and providing support to participants. | Behavior changes as a social diffusion process where norms shift through peer influence. Behavior change initiated by a few individuals’ spreads to others through network interactions and norm changes. | Increased HIV prevention advocacy, stigma reduction, HIV disclosure.  Marginally reduced condomless sex among social network members | Addresses internalized stigma but limited in assessing masculinity | Need for more tailored interventions for different age groups and cultural contexts. | The intervention was well-received, with high session attendance (76%), and showed promise in empowering individuals to advocate for HIV prevention. |
| 3 | (Rewley et al., 2020)  Tanzania | RCT | Change Agents in social networks | Interpersonal interactions facilitated by Change Agents | Change Agents recruited from HIV treatment facility waiting rooms | Emphasizes behavior change via knowledge-sharing by peers | 14.2% increase in HIV knowledge among network members | Potential spillover effects of highly connected individuals in peer networks. | Insufficient data on behavioral outcomes of peer network interventions | Effective in reaching network members, enhancing knowledge through interactions. |
| 4 | (Kwena et al., 2017)  Kenya | Cross-sectional | Fishermen | Community-based approaches to address contextual risks and foster behavior. | Focus group discussions and in-depth interviews guides focusing on fishing communities involved in the sex-for-fish economy | Encouraging behavior change through open communication and spousal support. | Identified risks in extramarital partnerships, recommended spousal communication. | Contextual risk factors tied to financial and socio-physical environments. | Peer interventions tailored for rural fishing communities | Interventions focused on community education, improved spousal communication, and individual self-evaluation for behavior change. |
| 5 | (Mangombe et al., 2020)  Zimbabwe | Cohort | Young people and adolescents | Peer education with professional and peer-to-peer network models | Peer education as a communication channel for health messages | Effective peer communication among young people; holistic integration with other interventions | Increased HIV testing, mental well-being, entrepreneurial skills | Lacked direct focus on masculinity barriers, stigma reduction | Under-researched on peer influence in adolescent care | Professional peer navigators and social network-based peer-to-peer approaches are acceptable and valued |
| 6 | (Ludwig-Barron et al., 2021)  Kenya, | Cross-sectional | Persons who inject with drugs (PWID) | Peer support, navigation services through peer educators to address barriers in HIV and Hepatitis C care for | Modified Social Ecological Model; Focus on urban setting, with mention of rural healthcare challenges | Peer networks offer essential support and navigation services to overcome access barriers. | Increased care access, reduced stigma and social isolation | Stigma, social isolation, and interactions with drug dealers, mental health challenges, and limited access to rural healthcare. | Specific needs of PWID populations, particularly in rural healthcare settings remain sunder explored. | Community-level interventions and peer support were highly valued. |
| 7 | (Katz et al., 2022)  South Africa | RCT | PLHIV | Treatment Ambassador Program | Client-centered counseling and patient navigation skills for people with HIV not on ART treatment. | Peer-driven support for ART barriers | Increased engagement among ART-delayed patients. High effectiveness in reaching vulnerable populations, providing feasible and acceptable support | No specific masculinity/stigma focus | Effectiveness in the urban community | Effective in engaging the disenfranchised populations by integrating peer support within community, maintaining high fidelity |
| 8 | (Marta et al., 2018), Tanzania | RCT | Peer-nominated health leaders | Network position-based peer education on HIV/GBV | Influence of network structure on engagement Focusing specifically on how leaders' roles within these networks shaped their social influence and effectiveness in promoting health discussions. | Network positions influence the effectiveness of peer-based interventions. Those with gatekeeping roles may be more successful in discussing sensitive health issues. | Central positions hinder sensitive topic discussions. Higher in-degree centrality was associated with health leaders reporting fewer conversations about HIV. Higher betweenness centrality led to more discussions on gender-based violence. | Influence of network roles on behavior. suggesting Gatekeepers could more effectively engage peers on controversial topics. | Influence of network structure on health behavior. Lack of direct assessment of ongoing support or training. | Popularity may hinder discussions on sensitive topics due to social pressures, while individuals in intermediary positions are more effective in driving discussions around gender-based violence, |
| 9 | (Brashers et al., 2017)  Kenya | Longitudinal study | PLHIV | One-on-one peer mentor support on managing illness, disclosure and enhancing social support. | Culturally relevant communication strategies to help PLHIV disclose their condition, seek social support, engage with healthcare providers. | Peer-led support systems that provide newly diagnosed individuals with tools to manage uncertainty, improve social connections, and develop effective communication strategies | Reduced illness-related uncertainty. Increased access to and satisfaction with social support. Decreased depressive symptoms. Enhanced psychosocial functioning Improved patient self-advocacy | Enhancing skills for disclosing HIV status and building social support networks | Need for further exploration of culturally adapted interventions for uncertainty management; limited by peer support duration | Peer mentors play a vital role in reducing uncertainty and improving psychosocial outcomes for newly diagnosed PLHIV by offering relatable, experience-based support |
| 10 | (Rabie et al., 2020)  South African | RCT | Men in soccer and vocational activities | Peer-based soccer and vocational training as HIV prevention contexts | Socio-economic and educational factors influencing outcomes. Male-centered, activity-based engagement incorporated through soccer and vocational training group activities. | Peer-based program leveraging popular community activities to engage men in HIV prevention. Familiar and interest-based activities may help optimize adherence and consistent participation in HIV prevention among men. | Higher adherence, engagement in high-risk men | Limited cultural factors exploration | Effect of community sports-based interventions on long-term HIV prevention outcomes | Interventions centered on sports and vocational training can engage high-risk men in HIV prevention, with certain socioeconomic and personal history factors influencing adherence. |
| 11 | (Ross et al., 2016)  Tanzania | Cross-sectional | MSM | Respondent-driven sampling-based recruitment for MSM HIV/STI networks | Utilized respondent-driven sampling to explore MSM networks for health intervention strategies | MSM social networks, particularly those including sexual partners, close friends, or acquaintances, could facilitate peer-based health interventions | High social connections for MSM, but limited reach to "loners" | Limited to scope to respondent-driven sampling. No intervention outcome data | Impact of Respondent-driven sampling criminalized population.  Research on social networks of "loner" | Respondent-driven sampling-based may overlook those not well-connected within MSM networks. |
| 12 | (Chime et al., 2018)  Nigeria | Cross-sectional | PLHIV | Peer support groups for ART adherence | Peer network interventions promote medication adherence, with group support as a key strategy | Peer networks enhance ART adherence in resource-limited settings | Peer support groups showed 91.9% adherence vs. 87.1% in non-support groups. Factors like alcohol intake and poverty were identified as barriers. | Alcohol intake, poverty, distance to health facilities | Need for further research on socio-economic barriers specific to resource-limited settings | Peer support groups are effective for improving ART adherence in men with HIV. |
| 13 | (Jemmott et al., 2014)  South Africa | RCT | Men in sexual risk-reduction program | Peer-based intervention for condom use and HIV/STI prevention | Targeted to men who have intercourse with women, focused on increasing condom use and reducing HIV/STI risk. | Peer networks support behavior change in sexual health risk reduction | Increased consistent condom use and safer sexual practices | No effect on unprotected intercourse or multiple partnerships | Lack of cultural tailoring in some areas | Condom use increased, but unprotected intercourse remained unchanged. |
| 14 | (Barnabas et al., 2022)  South Africa | RCT | HIV-positive individuals in low-income areas | Home delivery and monitoring of ART, with a fee-based option | Economic consideration for user-fee service delivery to assess affordability and acceptability in resource-limited settings | Fee-based home ART delivery can improve access in low-income, high-unemployment contexts | Significant increase in viral suppression among men from 64% to 84% with fee-based home delivery option | Economic feasibility in low-income settings | Long-term sustainability of fee-based services | High willingness to pay for the user fee in this setting suggests potential for similar differentiated service delivery models in other low-income, high-unemployment areas. |
| 15 | (Njau et al., 2023)  Tanzania | Longitudinal study | Male Mountain Climbing Porters | Behavior changes models for HIVST promotion on Hard-to-reach male porters | Hard-to-reach populations, Mountain climbing porters through Satisfaction-based training and support | Peer networks optimize HIV services by addressing hard-to-reach populations | Increased HIVST uptake among hard-to-reach populations.  High satisfaction with intervention sessions (rated 4.3 to 4.8 on satisfaction scale) | Environmental and occupational barriers and lack of sustained engagement strategies for hard-to-reach populations | Long-term behavior change engagement | High satisfaction and adherence scores |
| 16 | (Abu-Ba’are et al., 2023)  Ghana | Cross-sectional | GBMSM (Gay, Bisexual, and Other Men who have Sex with Men) | Peer educators involved in promoting HIV prevention strategies, focusing on access and awareness of HIV prevention strategies | Peer educator involvement | Peer networks facilitate HIV prevention strategies in stigmatized communities | Increased condom uses and PrEP uptake, though access and stigma remain barriers. | Limited access to healthcare facilities, stigma, and discrimination | Need for culturally tailored interventions for GBMSM | Highlights peer education as effective but emphasizes the need for culturally specific HIV prevention strategies |
| 17 | (Eubanks et al., 2022)  Mali, Côte d’Ivoire, Burkina Faso, and Togo | Cohort | MSM | Outreach activities via community-based organizations and peer educators, tailored to MSM communities | Peer educators provided guidance and follow-up focused on PrEP use and adherence | Focused on addressing social and economic vulnerabilities affecting PrEP adherence | Increased PrEP adherence and acceptability among MSM, with correct adherence defined as four pills per week | Adherence challenges among economically marginalized MSM; social vulnerabilities remain a barrier | Further exploration of culturally sensitive support for economically/socially marginalized groups and long-term impacts on behavior change | PrEP adherence increased through peer-led community-based interventions in West Africa. |
| 18 | (Kalichman et al., 2013)  South Africa | RCT | Men at alcohol–HIV risk | Multi-level alcohol–HIV risk reduction intervention: Individual workshops and community activation in shebeens | Individual workshops and community-level activation focused on HIV risk reduction; Tailored to men who drink in shebeens; incorporates social and structural intervention levels | Multi-level interventions target individual and community risk. | Increased condom use, more HIV prevention discussions, stronger perceptions of safer sex norms. | Addressed risk behaviors linked to masculinity and drinking culture. | Limited long-term sustainability of behavior changes. | Interventions targeting men in drinking settings could reduce HIV/STI risks. |
| 19 | (Smith et al., 2021)  South Africa | RCT | Urban men | U=U (Undetectable= Untransmittable) intervention, peer-led HIV testing in high-traffic urban areas | Peer educators provided U=U messaging and facilitated HIV testing at mobile clinics | U=U messaging reassures men and supports testing without altering lifestyle perceptions. | Increased HIV testing uptake and higher yield of positive cases; reduced fear of testing positive. | Fear and stigma surrounding HIV testing; need for long-term behavioral data | Need for studies on sustained changes in HIV testing behavior and long-term outcomes | U=U messaging effective in reducing stigma and fear. |
| 20 | (Nelson et al., 2015)  Ghana | Cross-sectional | MSM | Network-individual-resource (NIR) model of HIV prevention: Autonomy support and cultural sensitivity in healthcare interactions: | Peer support and cultural sensitivity in healthcare settings | Peer networks enhance autonomy in healthcare, contributing to safer sex practices and HIV prevention | Increased condom use and healthcare autonomy, higher supportive norms within MSM communities | Stigma reduction through peer-provider support; need for more autonomy-supportive interventions | Need for culturally appropriate, autonomy-supportive interventions. | Healthcare provider support crucial for influencing behavior. |
| 21 | (Matovu, Mbita, et al., 2021)  Tanzania | Cross-sectional | Heterosexual men | HIV self-testing education and promotion through social media engagement and peer network distribution | Peer network leaders distributed HIV self-test kits and provided follow-up support | Social networks enhance HIV self-testing uptake and reduce barriers to accessing HIV testing | Increased willingness to distribute and receive self-test kits within close male friendships | Some hesitancy and logistical challenges with kit distribution and follow-up | Further research needed on social network influences in self-testing. | Positive attitudes towards HIV self-testing within social networks. |
| 22 | (Ky-Zerbo et al., 2022)  Côte d'Ivoire, Mali, and Senegal | Cross-sectional | MSM | Peer distribution, outreach within key populations, referral to obtain kits if unavailable | Peer educators explained kit use, result interpretation, need for confirmatory testing, supported users through videos, free hotline, and in-person support on request | Peer networks valuable for HIVST access and acceptance, high reach within high-risk groups | Effectiveness in reaching secondary users and promoting testing among partners and key population peers | Minimal adverse reactions; logistical challenges in distribution; some reports of physical abuse | Limited long-term data on the intervention's impact on behavior | Secondary HIVST users test relatively soon (within 2 days) after receiving kits |
| 23 | (Adeagbo et al., 2022)  South Africa | RCT | Professional peer navigator | Peer support, mobile health via social network interactions, sexual health discussions, HIVST distribution | Peer navigators trained and support provided for PrEP promotion and HIVST | Peer support crucial for HIV testing, disclosure, and treatment adherence | Increased comfort in discussing sexual health, improved testing rates | Comfort discussing sensitive topics with trained peers over traditional healthcare workers | Limited data on long-term adherence | Peer navigators were effective in improving sexual health discussions and testing uptake. |
| 24 | (Kra et al., 2021)  Côte d'Ivoire, Mali, and Senegal | Longitudinal study | MSM | Peer outreach, modified group activities, hygiene integration, increased social network use | Adapted during COVID-19 to maintain service with modified protocols | Peer adaptation crucial for continuous testing access during crises | Resilience in HIVST distribution during the pandemic, increased testing uptake post-COVID-19 | Need for longitudinal studies on long-term HIV transmission rates and behavior | Need for longitudinal studies on long-term HIV transmission rates and behavior change | Peer-led interventions were resilient and adapted to the pandemic, ensuring continued HIVST access. |
| 25 | (Kra et al., 2022)  Côte d’Ivoire, Mali, and Senegal | Cross-sectional | MSM | Peer and secondary distribution, focus on reaching underserved groups | Kit usage support, emphasis on reaching first-time testers | Secondary distribution effective in engaging underserved populations for first-time testing | High proportion of first-time testers reached, particularly among younger and lower-educated individuals | Challenges in engaging older or more educated groups for self-testing | Further studies on sustained testing behavior needed | Peer networks can effectively engage first-time testers in HIV self-testing |
| 26 | (Matovu, Nambuusi, et al., 2021)  Uganda | Cross-sectional | Fishing community | HIVST kit distribution, individual follow-up to encourage kit usage and ART initiation | Lay providers trained for distribution; support for correct use and follow-up with network members | Lay peer leaders increase uptake effectively within community settings | Lay peer leadership boosts HIVST uptake and ART initiation within community settings | Literacy challenges among some community members, some resistance to HIVST uptake | Need for additional resources to aid illiterate users | Lay peer leaders significantly improved HIVST uptake in fishing communities. |
| 27 | (Okoboi et al., 2020)  Uganda | Cross-sectional | MSM | Distribution of HIVST kits through MSM peer networks, linkage for confirmatory testing | Peer support for kit distribution and linkage to care | Peer networks effective in reaching underserved, high-risk populations | Higher testing among never-testers, increased diagnosis and linkage to care | Long-term data on intervention effectiveness needed | Need for more long-term data on effectiveness | Highlights potential of peer networks to enhance testing and linkage to care in MSM populations. |
| 28 | (Matovu, Bogart, et al., 2020)  Lake Victoria Shore of  Uganda | Cross-sectional | Fishing community | Peer-led oral HIV self-testing (HIVST) to hard-to-reach fishing communities | Training provided to peer leaders on distributing HIVST kits and linking to care; details on training duration or ongoing support not specified. | Peer-led model effective for hard-to-reach communities, indicating a strong preference for local peer leaders. | High feasibility and acceptability of peer-led HIVST, with 99.3% of kits distributed and 96.6% used, with high linkage to care. Effective for fisher-folk. | Not identified | Insights into sustainability of the peer-led model. | Peer-led HIVST shows strong outcomes in hard-to-reach fishing communities, especially fisherfolk. |
| 29 | (Choko et al., 2018)  Uganda | Longitudinal study | Fishing Community | Peer-based distribution of HIVST kits by male “seeds” to men in underserved fishing communities | Seeds recruited among patients and community members; no specific training details provided. | Peers are effective in promoting testing in underserved groups, although minor hostility was reported. | High uptake of self-tests, with a significant number of first-time testers; shows potential to bridge gender gaps in testing. | Hostility encountered by seeds | Positive peer feedback; seeds and recruits willing to recommend HIVST to others. | Secondary HIVST kit distribution is feasible but requires structured support for linkage to care. |
| 30 | (Sithole et al., 2022)  South Africa | Cross-sectional | PLHIV | HIVST kit distribution, social network engagement | Linkage to care post-distribution | Peer networks expand access but face linkage-to-care challenges. | Increased testing rates (66% kit usage, 42% from first-time testers), low linkage to care for those testing positive | Limited linkage to care post-testing. | No long-term tracking of linkage to care. | Secondary HIVST kit distribution feasible but requires support for linkage to care |
| 31 | (Chang et al., 2019)  Mashonaland East and Central,  Zimbabwe | RCT | MSM | Voucher-based self-testing, promotional messages; Urban vs. rural distribution, price sensitivity | Men who have never had an HIV test | Price is a major barrier; promotional messaging did not increase demand | Demand for self-testing, price and location sensitivity, pharmacy effectiveness in urban areas | Price sensitivity, emotional burden of testing | Effectiveness of messaging strategies | Study highlights price sensitivity as a barrier to testing and emphasizes the role of accessible distribution channels. |
| 32 | (Hayes et al., 2017)  Zambia and South Africa | RCT | Men 15–60 years | Door-to-door HIV testing, ART initiation | Community outreach intervention | Universal testing effective, slower ART linkage in men | High testing uptake, ART initiation increases from 44% to 61% | Slow linkage, lower coverage in youth | Long-term engagement methods | Highlighted high testing and ART success but needs faster linkage |
| 33 | (Lebelonyane et al., 2021)  Botswana | RCT | Men in general | Outreach activities on communities not reached by national programs | High HIV-prevalence communities not reached by national programs | Targeted outreach increases testing and ART adherence | 30% reduction in HIV incidence, increased ART and viral load suppression | Not identified | Long-term outcomes and expanded implementation | Strong impact of targeted outreach on testing, ART adherence, and incidence reduction in high-HIV-prevalence areas. |
| 34 | (Mukumbang, 2021)  South Africa | Case Study | PLHIV | Men with ART-friendly masculinity focus | Peer-to-peer with Adherence Clubs and Pharmacy Pick-Up | Social cohesion, stigma reduction, ART-friendly masculinity | Improved ART engagement, reduced stigma | Not identified | Engagement and masculinity's role in ART adherence | Highlights cohesion, stigma-free, and convenience for men |
| 35 | (Mabaya et al., 2022)  Zimbabwe | Longitudinal study | Peer educators for adolescent health | Health and circumcision education | Incentives, though reduced over time | Peer-driven programs effective with intrinsic motivators | High resilience and intrinsic motivation in peer educators, VMMC and HIV testing increase | Funding reduction | Incentive role in long-term engagement | Peer resilience even with reduced incentives |
| 36 | (Kamanga et al., 2021)  Zambia | Longitudinal study | MSM | Peer leaders involved in the Testing and linkage tracking | Leadership and accountability intervention | Stigma and discrimination in key populations | increase in HIV positivity, sustained testing gains | Stigma and discrimination in key populations | Long-term sustainability of leadership interventions | Successful in targeted engagement for key populations |
| 37 | (Bogart et al., 2023)**.**  Botswana | RCT | PLHIV | Clinic-based treatment partner intervention (Mopati) | Peer networks enhancing ART adherence and supporting treatment partners | Peer networks effectively enhance adherence and provide caregiver support | Increase in viral suppression rates and adherence, decrease in caregiver burden | Positive impacts on both patient and caregiver well-being | Scale-up feasibility in diverse contexts | Demonstrated positive impacts on patient adherence and caregiver well-being, showcasing the power of peer support networks. |
| 38 | (Mantell et al., 2019)  Zimbabwe | Cross-sectional | PLHIV | Community-based antiretroviral refill groups, support groups and information sharing | Open communication among HIV care recipients, community members, healthcare workers, donors, and policymakers | Community-based antiretroviral offered psychosocial support and facilitated engagement in HIV care. | Increased male engagement in ART refill groups and Efficiency, convenience, and psychosocial benefits; improved coping with HIV | Stigma, privacy concerns, and limited awareness | Male-specific barriers to engagement in HIV care | Community-based antiretroviral refill groups proved beneficial but require strategies to address stigma and privacy concerns. |
| 39 | (Kamya et al., 2021)  Uganda and Kenya | RCT | PLHIV | Focus on HIV testing and ART initiation for men with low CD4 | Community-based, patient-centered care model | Streamlined care as a model for reducing mortality | Increased ART initiation and reduction in mortality | Non identified | Nono identified |  |
| 40 | (Kelvin et al., 2021)  Kenya | RCT | Truck drivers | HIVST outreach activity | HIVST intervention for truck drivers | Limited effectiveness of HIVST in truck drivers | Increased HIV testing rates and preferences for testing methods | Low perceived risk, lack of time, and fear of testing | More effective HIVST delivery models tailored to truck drivers; preference for blood-based tests | Preference for blood-based tests |
| 41 | (Yamanis et al., 2017)  Tanzania | Cross-sectional | Peer to Peer | Social Media Engagement/ Network: | Network structure, composition, norms related to men’s HIV testing | Peer networks facilitate testing by reducing stigma and normalizing testing | Increased likelihood of testing among network core members; stigma reduced testing rates. | Limited data on long-term efficacy | Gaps in understanding the effectiveness of sustained social media engagement |  |
| 42 | (Conserve et al., 2019)  Tanzania | Cross-sectional | Peer to Peer | Peer-enablers and barriers of HIV testing among men | National strategies for improving testing uptake | Peer networks provide encouragement and reduce barriers through social and cultural approaches. | Increased testing via encouragement from partners and awareness. | Addressed fear of positive results, low risk perception. | Limited data on long-term efficacy. | Multi-pronged strategies addressed barriers to testing. |
| 43 | (Rotsaert et al., 2022)  Zimbabwe | Longitudinal study | HIVST distribution campaign | Community-based HIVST distribution | Targeted to uneducated, rural, and younger demographics | Peer networks (household heads) impacted distribution and awareness. | Effectiveness: Identified low awareness of HIVST in key demographics. | Limited awareness among younger, less-educated individuals. | Gaps in reaching low-awareness groups. | Need for tailored HIVST distribution to increase awareness. |
| 44 | (Hensen et al., 2015)  Zambia | RCT | Men in general | HIV-testing behaviors among men 15–60 years in rural districts | Highlights need for alternative testing methods like self-testing and mobile testing to increase rates | \| Factors influencing HIV testing; acceptance of home-based testing \| \| --- \|  \|  \| \| --- \| | Increased testing behavior | Need for alternative strategies to increase testing in high prevalence | Current testing methods insufficient; suggests need for alternative strategies |  |
| 45 | (Makhema et al., 2020)  Botswana | RCT | Outreach Workers | Community-wide HIV testing campaign: combination prevention project | Home-based and mobile HIV testing, facility enhancement, outreach | Describes community-based HIV testing to maximize linkage to care and ART | Increase in HIV-positive participants with viral suppression; higher male circumcision rates | No significant barriers reported | Not highlighted |  |
| 46 | (Ndungu et al., 2023)  Kenya | Cross-sectional | MSM | HIVST kits and immediate linkage to care and Factors influencing HIVST among MSM | Focus on self/partner care awareness | Interconnectedness of various factors in HIVST uptake | Increased HIVST acceptability and uptake | Social stigma among MSM not addressed | Motivators for self-testing, especially among different socioeconomic groups | Need to explore stigma mitigation strategies |
| 47 | (Matovu, Nambuusi, et al., 2020)  Tanzania | Cross-sectional | Peer to Peer | Part of larger study on HIVST intervention | Peer-led HIV self-testing | Familiarity with peer leaders facilitates access to testing services | Increased testing access via peer leaders | Fear of positive results and need for testing support | Lack of data on support during testing | Need for support mechanisms post-testing |
| 48 | (Tanser et al., 2021)  South Africa | Cross-sectional | Peer to Peer | Home-based testing with small incentives, decision support app | Financial incentives as motivators | Testing motivations and behavior via incentives and decision apps | Increased uptake of HIV testing by 50% with financial incentives | Social norms and masculinity-related barriers not addressed | Understanding internal vs. external motivators for testing | Further research on decision apps |
| 49 | (Ndyabakira et al., 2019)  Uganda | RCT | Incentive-based CH | Gain, loss, and lottery incentives for testing; Testing accessible in public, high-mobility locations | Public incentives reducing stigma | Stigma reduction through social proof for testing. Incentives reduce costs and stigma; create "social proof" for testing | Increased testing and reduced barriers | Social stigma addressed through public incentive provision | Sustainability of incentives for long-term use |  |
| 50 | (Mantell et al., 2022)  Kenya | RCT | Truck drivers | Oral and blood-based HIVST kits, choice-based testing | Testing preference among truck drivers | Preference for oral vs. blood-based testing for increased uptake | Increased testing with choice-based intervention | Stigma around HIVST kits not addressed | Need for more research on HIVST options and barriers |  |
| 51 | (MacCarthy et al., 2020)  Uganda | RCT | Peer to Peer | Focused on personalized and peer adherence messaging. | Text-based intervention focused on adherence messages. | Peer Network Interventions described as peer-influenced adherence messaging. Peer adherence information positively influenced participants. | T1 (own adherence messages) led to a 3.8% decrease in adherence. T2 (own and peers' adherence messages) initially increased adherence by 2.4%, with a 9.0% increase by study end. | Text based intervention (T1) less effective for sustained adherence | Impact of peer messaging on long-term adherence | T2 provided a growing positive impact over time, suggesting potential for peer influence in adherence behavior. |
| 52 | (Dulli et al., 2020)  Nigeria | RCT | PLHIV | Social Media Engagement/ Network: | Social media platform for adherence, retention, and support. | Knowledge enhancement through social media platforms | \| Improved HIV knowledge, but no significant retention impact \| \| --- \|  \|  \| \| --- \| | No measurable effect on retention or social support | Further research needed on social media peer support and its impact on retention. | Positive feedback from participants on web-based platform usability and acceptability. |
| 53 | (Lippman et al., 2023)  South Africa | RCT | Peer to peer | SMS and SMS with peer navigation for linkage to care | Peer navigation for better linkage and ART initiation | Comparison of SMS-only and SMS + Peer network interventions | SMS participants linked at 1.28 times the rate of the standard care; SMS+PN participants linked at 1.60 times the rate. SMS+PN improved ART initiation time. | Balancing intervention intensity based on client needs | Need to explore the intensity of interventions | SMS+PN doubled linkage rates, highlighting the potential for scale-up. |
| 54 | (Daniels et al., 2023)  South Africa | Cross-sectional | MSM | Social Media Engagement/ Network: | Healthy Relationships intervention adapted into SOAR videoconference format and Conducted via Zoom for HIV status disclosure and relationship dynamics. | Peer Network Interventions seen as tools for HIV status disclosure. Success factors: Videoconference usability and participant satisfaction. Limitations: Only short-term satisfaction data available. | High satisfaction and usability, with 69% rating the experience as good and 31% as excellent. |  | Need for feasibility testing in videoconferencing contexts. | SOAR will be further tested in a pilot RCT. |
| 55 | (Kuhns et al., 2021)  Nigeria | Cross-sectional | MSM | mHealth and SMS for HIV engagement | iCARE Nigeria peer navigation and mHealth and social media usage in Nigeria; Platforms like Facebook and Grindr; Designed to minimize data usage. | Focus on data-efficient strategies for local platforms | High acceptability and positive perceptions; feasibility adaptations for mHealth to maximize reach and safety. | Addressed context-specific barriers in digital engagement. | Long-term feasibility and safety in mHealth not fully understood. | Adapted social media and mHealth to local user needs. |
| 56 | (Abubakari et al., 2021)  Ghana | Longitudinal study | MSM | ADAPT-ITT model to modify a smartphone-based peer support intervention, | Peers trained in emotional, informational, and appraisal support. | Adaptation of culturally relevant communication. Limitations: Social stigma and access limitations. | Culturally relevant peer support improved prevention and care uptake. reduced intersectional stigma and enhance HIV health-seeking behaviors among MSM. | Addressed intersectional stigma and barriers to accessing services. | Impact of intersectional stigma on service uptake. | Effective in recruitment for HIV testing and counseling services among MSM. |
| 57 | (Takada et al., 2020)  Mbarara, Uganda | Cross-sectional | Peer to Peer | Social Media Engagement/ Network: | Sociocentric social network; Interpersonal interactions, verbal communication | Peer networks' role in shaping stigma, with ties to people living with HIV reducing personal stigma. | Peer networks influence individuals' stigma beliefs. Higher stigma scores in one’s social network are associated with higher personal stigma; having HIV-positive ties is linked to lower stigma. | Lower stigma among individuals with HIV-positive ties | Under-researched area on how social ties impact stigma. |  |
| 58 | (Verinumbe et al., 2023)  Nigeria | Cross-sectional | PLHIV | Group Discussions focused on addressing stigma mitigation | Focus on internalized stigma in people living with HIV (PLHIV) | peer support groups as effective in reducing stigma and addressing barriers to sustained HIV treatment. | Engagement in peer support groups appeared to mitigate internalized stigma among PLHIV. | Internalized stigma | Not identified | Highlights the importance of peer support in combating internalized stigma. |
| 59 | (Graham et al., 2020)  Kenya | RCT | PLHIV | Individual counseling, peer matching, support counseling | Nurse-led counseling and peer support for PLHIV | Peer support linked to higher retention and treatment adherence, with increased viral suppression through increased social and emotional support. | Intervention led to improved ART adherence and viral suppression | Stigma and discrimination; gender-specific barriers to retention and adherence | Not identified | Emphasizes nurse-led and peer-matched support for better |
| 60 | (Heslop & Banda, 2013)  Eastern Zambia | Longitudinal study | Peer to Peer | Culturally contextual discussions among young people | Participatory peer approach, though training details not specified | Peer discourse influences young people’s perspectives on HIV prevention. | Highlights complex discourse on sexuality and HIV, emphasizing a need for tailored prevention approaches. | Cultural norms and limited safe consensual sex opportunities | Under-researched socio-cultural factors in sexual decision-making | Inclusion of traditional and religious leaders helped align social norms with HIV prevention. |
| 61 | (Maina et al., 2018)  Ghana | Case Study | MSM | Peer led discussion | Analyzes social network structure and function for support and refuge among MSM | Peer networks provide essential support and act as places of refuge. | Networks provide psychological support and function as a refuge; organized through informal rules. | Highlights masculinity-related barriers and need for secure peer support | Limited research on social network structure and function among MSM in varied contexts | Emphasizes roles of group leaders, with qualities like financial stability and experience. |
| 62 | (Sileo et al., 2021)  Uganda, | Cross-sectional | Fishermen | Outreach activities targeting fisherfolk | Peer network for fisherfolk addressing gender norms and HIV stigma's impact on treatment adherence | Masculine norms and stigma influence adherence; peer networks support engagement. | Gender norms and stigma associated with missed HIV visits and ART adherence. | Stigma and masculinity-related adherence barriers | Not idented |  |
| 63 | (Fawzi et al., 2019)  Tanzania | RCT | Change agents | NAMWEZA intervention focused on HIV prevention, sexual, and reproductive health. | HIV-positive individuals were empowered as change agents, enhancing community-based prevention efforts. | HIV-positive individuals can effectively serve as change agents, reducing stigma and promoting safer practices. | 40% decrease in IPV among women; improved self-efficacy for safer sex. | Limited behavioral change among social network members for HIV risk behaviors. | Not idented | Peer network interventions reduced stigma, IPV, and depression while enhancing self-efficacy. |
| 64 | (Denison et al., 2020)  Ndola, Zambia | RCT | Youth peer mentors | \| Training and employing HIV-positive youth as clinic-based mentors through Project YES \| \| --- \|  \|  \| \| --- \| | Youth peer mentors received proper training and were compensated as clinic-based staff. | Youth peer mentors serve as an effective, feasible approach for adolescent engagement. | Improvement in ART adherence, decrease in internalized stigma, increase in viral suppression. | Addressed stigma and provided adolescent-centered support. | Not idented | Youth mentors played a key role in achieving viral suppression and reducing stigma among HIV-positive adolescents. |
| 65 | (Pettifor et al., 2018)  South Africa | RCT | \| local community mobilizers \| \| --- \|  \|  \| \| --- \| | Community mobilization intervention, addressing gender norms among rural men | Activities led by local mobilizers and volunteer Community Action Teams. | Peer networks effectively addressed harmful gender norms, promoting equitable practices. | Improved gender norms among men exposed to the intervention; however, no immediate changes in risk behaviors. | Addressed masculinity-related barriers. | Indicated that longer intervention periods may be needed for behavior change. | Community mobilization showed promise in changing gender norms but required continued intervention. |
| 66 | (Dramé et al., 2013)  Senegal | RCT | MSM | Awareness campaigns and community engagement events | Awareness campaigns and community engagement activities | Men with confidants showed reduced risky behaviors, emphasizing the role of trust and social capital. | Reduced HIV and STI prevalence, lower risk behaviors, increased social capital | Loss to follow-up due to death, relocation, or loss of contact. | Not idented | Awareness campaigns enhanced social networks and reduced risky behaviors. |
| 67 | (Naidoo & Johnson, 2013)  Namibia | Longitudinal study | Men in the general population | Community-based natural resource management (conservancy program) in Rural communal areas | Outreach activities; community workshops; digital platforms for Treatment as Prevention information | Conservancy programs integrated HIV prevention with community-based initiatives, reducing risky sexual behaviors. | Decrease in multiple sexual partnerships among men (50% reduction) with lower infections in communal areas. | Not idented | Further exploration of long-term sustainability and scalability. | Combining HIV prevention with community programs showed promising outcomes. |
| 68 | (Kabami et al., 2017)  Uganda, | Cross-sectional | Rural, resource-limited communities | Community-led multi-disease health campaign | Campaigns led by community leaders, volunteer health teams, and Ministry of Health staff. | Community-led health campaigns demonstrated effectiveness in scaling up testing and preventive services rapidly. | Successfully reached over half of a rural community in scaling up HIV testing and other preventive services. | Not idented | \| Scaling to urban areas or different cultural contexts. \| \| --- \|  \|  \| \| --- \| | Community-led approach proved effective in rapid scale-up of health services. |
| 69 | (An et al., 2022)  South Africa | RCT | PLHIV | Group-based behavioral HIV care continuum | Peer-facilitation with non-judgmental support for men transitioning from incarceration to community | Peer support facilitated adherence by sharing experiences and providing mutual encouragement. | Improved ART adherence, life skills, and overall engagement in care | Transportation issues, illicit drug use among members, and social influences. | Post-incarceration needs and systemic barriers faced require tailored solutions. | Peer-led approaches showed high value in supporting transitions and improving adherence. |
| 70 | (Ibiloye et al., 2023)  Benue State, Nigeria, | Cross-sectional | MSM | Peer-to-peer approach (Community-Based ART delivery, CBART) | Counseling, patience, empathy, and tailored support to client context | Disclosure supports improved trust, adherence, and retention by providing safety and minimizing negative outcomes. | Improved adherence and care retention in a secure, supportive environment | Stigma, legal challenges, harassment, and discrimination. | Legal and contextual barriers affecting key populations | Inclusion of key populations in HIV service design improved care outcomes and medication adherence. |
| 71 | (Lukyamuzi et al., 2023)  Uganda | Cross-sectional | PLHIV | Community Health Workers (CHW) support HIV disclosure among PLHIV with disclosure challenges. | Ongoing support: Regular counseling and training for CHWs to aid in effective HIV disclosure. | CHW-led support fostered trust and offered tailored approaches, enhancing disclosure success. | Improved disclosure support, highlighting its role in treatment adherence | Confidentiality concerns and stigma fears limited disclosure. | Limited understanding of cultural preferences for disclosure, need for targeted stigma mitigation training. | CHW-led interventions are effective but require cultural adaptation and specificity in training. |
| 72 | (Lillie et al., 2019)  Burundi, Cote d’Ivoire and DRC | Longitudinal study | MSM | Targeted outreach campaign to reach new HIV-positive cases among key populations. | Enhanced Peer Outreach Approach (EPOA), targeting HIV services in key populations. | Enhanced Peer Outreach Approach is effective in reaching unreached populations, emphasizing the potential of peer-driven, time-bound outreach. | Higher HIV positivity detection rates, indicating success in reaching high-risk individuals | Limited assessment of long-term sustainability and impact over extended periods. | \| Insufficient data on cultural adaptability and campaign sustainability beyond its active period. \| \| --- \| | Highlights the importance of peer-driven interventions in enhancing HIV service access among key populations; sustainability strategies need exploration. |
| 73 | (Mulawa et al., 2018).  Dar es Salaam, Tanzania | Longitudinal study | Peer-nominated health leaders | Peer-nominated health leaders with microfinance integration for intervention support. | Knowledge and communication skills training on HIV and gender-based violence topics; Community members selected as peer leaders | Peer influence and structural position within networks are crucial for engaging peers on sensitive issues. | Positive impact on HIV and gender-based violence discussions; leaders with high "betweenness" centrality showed greater effectiveness. | Indirectly addressed by focusing on non-judgmental leadership. | More understanding needed on the role of structural network positions in peer influence effectiveness. | Long-term outcomes were not specified; microfinance component may support sustainability but was not directly assessed. |
| 74 | (Maman et al., 2020)  Dar es Salaam, Tanzania | RCT | Young men in social network camps | Training combined microfinance with peer health leadership | Young men’s social network “camps” focused on microfinance and peer health leadership. | Focused on using peer influence and social networks to promote gender-equitable attitudes and HIV testing. | Increased HIV testing rates and reduced inequitable gender norms among participants. | Discussions and addressing attitudes toward gender norms. | Limited strategies for engaging men in both HIV and intimate partner violence prevention. |  |
| 75 | (Van der Elst et al., 2020)  Kilifi,  Kenya | Cross-sectional | MSM | Engagement with policy leaders, health facilities, and community leaders | Organized response to improve HIV services through a decentralized health system | Peer networks facilitated collaboration and policy engagement to support MSM healthcare needs. | Improved HIV service access and community engagement for MSM. | Addressed through community and policy engagement. | Need for more research on decentralized, culturally tailored healthcare for MSM. |  |
