# Supplementary material for: Peer network approaches for improving HIV testing, prevention and care utilisation among men in sub-Saharan Africa: a scoping review
Source: BMJ Open. 2026 Mar 12;16(3):e106124. doi: 10.1136/bmjopen-2025-106124 (PMC12983769; doi:10.1136/bmjopen-2025-106124)
Supplement: Supplementary file 2 [file bmjopen-2025-106124.reviewer_comments.pdf]

PEER REVIEW HISTORY

BMJ Open publishes all reviews undertaken for accepted manuscripts. Reviewers are asked to complete a checklist review form and are provided with free text boxes to elaborate on their assessment. These free text comments are reproduced below.

ARTICLE DETAILS

Title (Provisional)

Peer network approaches for improving HIV testing, prevention and care utilization among men in sub-Saharan Africa: A Scoping Review

Authors

Otambo, Wilfred Ouma; Harling, Guy; Inghels, Maxime; Otto, Margot; Blose, Ntombifuthi; Tram, Khai Hoan; Tanser, Frank; Mee, Paul

---

VERSION 1 - REVIEW

**Reviewer**	**1**
**Name**	**Waweru Mwaniki, Samuel**
**Affiliation**	**School of Clinical Medicine, Faculty of Health Sciences, University of the Witwatersrand, Johannesburg, Wits RHI**
**Date**	**01-Nov-2025**
**COI**	**None**

Limitations: The limitations discussed here appear to be the limitations of the peer network interventions. However, limitations as envisaged in the PRSIMA (ScR) guidelines are limitations of the scoping review process. Please address this issue. For instance, limitation could include exclusion of papers published in other languages than English, and the fact that quality appraisal for the studies was not done etc.

Peer network approaches for optimized HIV prevention and care access among men in Sub-Saharan Africa: a scoping review

Thank you for the opportunity to review this manuscript. It addresses an important issue – that of investigating peer network approaches for optimizing HIV prevention, care and treatment for men in SSA, who lag behind in various UNAIDS targets.

Please see below some suggestions to improve the quality of the work:

Introduction:

The acceptable terminology for men who have sex with men is “gay, bisexual and other men who have sex with men” (GBMSM), although the authors can choose to state that this is abbreviated as MSM in their manuscript. Please also check throughout the manuscript for

instances where the term “men having sex with men” is used instead of “men who have sex with men”.

Methods:

Framework for conducting the scoping review: Although the authors have shown the “Analytical Framework for Network Interventions in the HIV Care Cascade” that they used, I suggest indicating which framework for scoping reviews they anchored their work on. Examples of these frameworks e.g. Arksey and O’Malley, appear as footnotes in the PRISMA (ScR) checklist and the authors could review them and align their work appropriately.

Inclusion and exclusion criteria: Please indicate if language was considered. For instance, did the authors retrieve papers published in English, or were other languages considered too? This is important as it has implications for Francophone SSA countries, meaning that if French was not considered, then publications from such countries may have been excluded.

Protocol registration: In addition to the registration number on PROPSERO, please consider providing a web address from which the protocol can easily be accessed.

Data charting process: Please indicate whether the form used was designed and pre-tested specifically for this study (or other similar studies), and by whom – the researchers in the current study or other researchers.

Quality assessment: in the term Preferred Reporting Items for Systematic Reviews and Meta-Analyses (PRISMA-ScR) guidelines, please add “extension for scoping reviews” before the abbreviation.

Results:

For Table 1, under the column ‘Description’, consider aligning text to the left (instead of justify) to improve readability.

For Table 2, consider combining the two target populations of PLHIV. Although the second group is explicit is for PLHIV with disclosure challenges, the interventions and outcomes do not seem to be very different for both groups, so combining would make the two groups would make the tale more concise.

Also consider the same for the MSM and GBMSM groups in the same table.

Under the “Peer Network Approaches and HIV Care Outcomes” sub-section, although this is a minor issue, please check and arrange the references chronologically when writing about several issues in a sentence e.g.

These include peer education sessions for prevention in Uganda [17], enhancing HIV knowledge and self-testing uptake among hard-to-reach populations in Tanzania [37], behavior change among fishermen in Kenya [32], and improving medication adherence and viral suppression in Nigeria [38] and South Africa [46].

Ideally should be 17,32,37,38, 46.

Also check in other parts of the manuscript where this issue occurs.

Discussion:

Limitations: The limitations discussed here appear to be the limitations of the peer network interventions. However, limitations as envisaged in the PRSIMA (ScR) guidelines are limitations of the scoping review process. Please address this issue. For instance, limitation could include exclusion of papers published in other languages than English, and the fact that quality appraisal for the studies was not done etc.

Minor issues:

Please ensure consistency in the various levels of sub-heading. Upper and lower cases are not used consistently.

Reviewer **2**

Name **Segawa, Ivan**

Affiliation **Makerere University College of Health Sciences, Clinical Epidemiology Unit**

Date **16-Dec-2025**

COI **None**

OVERALL COMMENTS:

The review is well written and exhausts the subject matter. I have a few concerns to address.

TITLE:

Small edit: ... sub-Saharan Africa: A scoping review (sub in lower case and A in upper). Perhaps we could include HIV testing (HIV testing, prevention, and care). I am not sure that "optimized" (improve?) and "access" (utilization?) are the right choice of words. Could we call them men at high risk of HIV acquisition or men belonging to key and priority populations?

INTRODUCTION:

Is limited male engagement confined to sub-Saharan Africa, or is it a global problem that is particularly pronounced in this region? Please clarify what you mean by this statement: "The current study will address this gap to the need to map the broadness of peer network interventions..."

METHODS:

Search strategy and information sources: Update the name of the subheading to include information sources. Did you search any gray literature (study registers, regulatory databases, websites, conference proceedings, and other online repositories)?

Inclusion criteria: It may be better to call inclusion and exclusion eligibility criteria. Why November 2013? You state that you wanted recent articles, but I'm not sure 2013 qualifies as recent.

Exclusion criteria: This should not be the opposite of the inclusion criteria. Also, were all articles in English, and was language not part of the criteria?

Screening and selection: How many people screened the articles or used the software? Also, describe how Rayyan was used (e.g., to remove records or replace a single screener) and trained (if relevant), and what internal or external validation was done to understand the risk of missed studies or incorrect classifications.

RESULTS:

Table 3: Would it be possible to quantify some of the linked outcomes without making the table bigger?

DISCUSSION:

We can't confidently say peer approaches are "highly effective" because the evidence is based off clinical trials or systematic reviews. Perhaps we could use alternative terms. Readers could also benefit from quantifying the impact of some of the peer approaches throughout the discussion. For example, increase in HIV testing rates, knowledge, timely linkage to care, ART adherence, and retention.

Limitations: Discuss any limitations of the evidence included in the review. Discuss any limitations of the review processes used, and comment on the potential impact of each limitation. These should be limitations of the review, not of the approach. You can leave that text without a subheading and create another for limitations

---

VERSION 1 - AUTHOR RESPONSE

Comments from Reviewers	Author Response	Ref.
**REVIEWER 1**		
The limitations discussed appear to be limitations of the peer network interventions rather than limitations of the scoping review process as per PRISMA-ScR.	Thank you for taking time to review the manuscript. The limitations section has been fully revised to focus strictly on limitations of the scoping review process, including language restriction, exclusion of grey literature, absence of formal quality appraisal, and heterogeneity of evidence. Limitations related to intervention design or implementation were relocated to a separate “Implementation Challenges” subsection.	Limitations of the Review Line 397-414<br><br>Implementation Challenges Line 415-428
**Introduction** Acceptable terminology is “gay, bisexual and other men who have sex with men (GBMSM)”; avoid “men having sex with men.”	Terminology was standardized throughout the manuscript to “gay, bisexual and other men who have sex with men (GBMSM),” with the abbreviation defined at first mention. All instances of non-standard phrasing (e.g., “men having sex with men”) were corrected for consistency and accuracy.	Throughout manuscript

**Methods**<br>Framework for conducting the scoping review: Specify which scoping review framework was used.	The Methods section states that the review was anchored on the Arksey and O'Malley framework, with refinements from Levac et al. and methodological guidance from the Joanna Briggs Institute. Reporting was aligned with the Preferred Reporting Items for Systematic Reviews and Meta-Analyses extension for scoping reviews (PRISMA-ScR).	Methods: Framework for Conducting the Scoping Review<br>Line 93-102
**Inclusion and exclusion criteria Language considerations:** Clarify whether language was considered and implications for Francophone SSA.	We have clarified that only English-language publications were included in the review. The potential exclusion of studies from Francophone and Lusophone sub-Saharan Africa is now explicitly acknowledged and discussed as a limitation of the review.	Methods: Eligibility Criteria; Line 120-134,<br><br>Line 400-402
**Protocol registration:** Provide a web address in addition to the PROSPERO registration number.	In addition to the PROSPERO registration number, a direct web link to the PROSPERO registry has been added<br><br>https://www.crd.york.ac.uk/PROSPERO/recorddashboard	Protocol Registration<br>Line 136-139
**Data charting process:** Clarify whether the form was designed and pre-tested, and by whom.	The manuscript now specifies that the data charting form was developed specifically for this study, pilot-tested by the study team, and refined iteratively. Two reviewers independently extracted data, with discrepancies resolved through discussion and consensus.	Data Charting Process<br>Line 153-156
**Quality assessment terminology:** Add "extension for scoping reviews" before PRISMA-ScR.	The terminology was corrected throughout the manuscript to "Preferred Reporting Items for Systematic Reviews and Meta-Analyses extension for scoping reviews (PRISMA-ScR)".	Line 96-99
**Results**<br>**Table 1:** Align text to the left in the "Description" column for readability.	Thank you. Table 1 has been revised so that all text in the "Description" column is left-aligned, improving readability and visual clarity.	Table 1
**Results**<br>**Table 2:** Consider combining PLHIV groups and MSM/GBMSM groups to improve conciseness.	Thank you for the guidance. The MSM and GBMSM categories were merged into a single group in Table 2 due to substantial overlap in intervention strategies and outcomes. This consolidation improves clarity while retaining population-specific nuances within descriptions.	Table 2
**Results**	Thank you. References cited within sentences were reordered chronologically (e.g., [17, 32, 37,	Line 284-304

**Reference ordering:** Arrange references chronologically within sentences.	38, 46]). A systematic check was conducted across the Results section and similar issues were corrected throughout the manuscript.	
**Limitations:** Limitations should reflect the scoping review process, not the interventions.	The Limitations section is now fully rewritten to focus exclusively on scoping review process limitations, including language restriction, exclusion of grey literature, lack of formal quality appraisal, and heterogeneity of included studies. Intervention-related issues have been moved to "Implementation Challenges" subsection.	Line 397-414,<br>Line 415-428
**Minor issues Subheading consistency:** Ensure consistent use of upper and lower case.	All headings and subheadings were reviewed and standardized for capitalization and hierarchy, ensuring consistency across sections in line with BMJ Open formatting conventions.	Throughout manuscript
**REVIEWER 2**		
**Title**<br>Adjust capitalization; include HIV testing; reconsider "optimized" and "access"; clarify population terminology.	The title has been revised to correct capitalization ("sub-Saharan Africa"), explicitly include HIV testing, replace "optimized" with "improving", replace "access" with "utilization", and clarify the focus on men at high risk of HIV acquisition, including key and priority populations.	Title page
**Introduction**<br>Is limited male engagement confined to SSA, or is it a global problem?	The introduction now clarifies that limited male engagement in HIV services is a global challenge, but one that is particularly pronounced in sub-Saharan Africa due to intersecting structural, social, and health-system constraints.	Introduction, Line 2-3<br><br>Line paragraph 54-66
Clarify the statement: "map the broadness of peer network interventions."	The sentence has been rewritten to clearly state that "the current review aims to systematically map the scope and characteristics of peer network interventions targeting men in SSA, describe the strategies employed, and identify reported opportunities and challenges across diverse contexts."	Introduction, line 79-84
**Methods**<br>Search strategy: Update subheading to include information sources. Did you search grey literature?	The subheading was revised to "Search Strategy and Information Sources." The manuscript now states that grey literature was not systematically searched, and the implications of this exclusion are addressed in the limitations.	Search Strategy and Information Sources Line 104, Line 113-117, line 403-407

**Methods: Inclusion criteria:** Rename criteria and justify November 2013 as the start date.	The section was renamed "Eligibility Criteria." The November 2013 cut-off was justified as corresponding with the scale-up of differentiated service delivery models, HIV self-testing, and peer/network-based HIV interventions in SSA.	Methods Eligibility Criteria Line 120 Line 124-127
**Methods: Exclusion criteria:** Should not simply mirror inclusion criteria. Was language part of the criteria?	Eligibility criteria were clarified to avoid inversion. The manuscript now explicitly states that only English-language publications were included, with the potential exclusion of Francophone and Lusophone SSA evidence acknowledged as a limitation.	Methods Eligibility Criteria Line 129-134
**Methods Screening and selection:** How many reviewers? How was Rayyan used and validated?	The manuscript now specifies that two independent reviewers conducted title/abstract and full-text screening. Rayyan was used for duplicate removal, blinded screening, and discrepancy identification. Disagreements were resolved through consensus, minimizing the risk of missed studies or misclassification.	Methods Screening and Selection Line 141-150
**Results Table 3:** Can some linked outcomes be quantified without expanding the table?	Table 3 was revised to incorporate concise quantitative descriptions within the outcome's column, without increasing table size.	Results Table 3
**Discussion:** Avoid stating peer approaches are "highly effective."	Language has been revised to more cautious, evidence-aligned terms such as promising, It now reads "Peer network approaches represent promising strategy for improving HIV prevention, treatment, and care among men in sub-Saharan Africa."	Discussion Line 343-346
Quantify impacts of peer approaches where possible.	Where primary studies reported quantitative outcomes, these were incorporated throughout the discussion, including increases in HIV testing uptake, improvements in linkage to care, ART adherence, and retention, while clearly attributing estimates to individual studies.	Discussion Line 346-356, Line 359-362, Line 373-380
**Limitations:** Discuss limitations of the evidence and of the review process; focus on review limitations.	The limitations section has been rewritten to focus on scoping review process limitations, with intervention-level issues moved to a separate Implementation Challenges section.	Discussion Limitations Line 397-414, Line 415-428

VERSION 2 - REVIEW

Reviewer 1

Name Waweru Mwaniki, Samuel

Affiliation School of Clinical Medicine, Faculty of Health Sciences, University of the Witwatersrand, Johannesburg, Wits RHI

Date 29-Jan-2026

COI

The authors have satisfactorily addressed all the comments raised in the first round of review. It is my view that the manuscript may now be accepted for publication in its current form.

Reviewer 2

Name Segawa, Ivan

Affiliation Makerere University College of Health Sciences, Clinical Epidemiology Unit

Date 10-Jan-2026

COI

The review comments have been sufficiently addressed. Just a minor concern about MSM in full. "Men who have sex with men" is more commonly used than "men having sex with men." I am not sure why the latter term was used, but I might be mistaken.
